# Integrating Kinetics into Synthetic Studies via Adaptable Concentration‐Dependent Yield Analysis

**DOI:** 10.1002/advs.77694

**Published:** 2026-09-27

**Authors:** Xinyi Xiao, Koki Ikemoto, Toshiya M. Fukunaga, Hideaki Nishina, Hiroyuki Isobe

**Affiliations:** ^1^ Department of Chemistry The University of Tokyo Bunkyo‐ku Tokyo Japan

**Keywords:** concentration‐dependent yield analysis, kinetics, macrocycles, organic synthesis, template synthesis

## Abstract

When inversion of optical rotation was discovered during the hydrolysis of sucrose, the name “invert sugar” was coined for this sweetener. The inversion process sparked the field of chemical kinetics by introducing time‐course analyses and differential rate equations as important tools for studying reaction mechanisms and rates. These tools have become indispensable in kinetic studies and have also advanced other fields, including biochemistry and the chemical industry. In this study, chemical kinetics are integrated into synthetic studies. The modeling approaches in this study, i.e., machine‐learning (ML) modeling of yield distributions and Euler‐integration‐based modeling of kinetics, are found to be sufficiently versatile and adaptable to accommodate chemical and operational considerations, allowing concentration‐dependent yield analysis (CYAN) to reveal kinetics hidden behind synthetic yield data. Importantly, the ML+CYAN approach reveals unexpected aspects of metal‐templated oligomeric macrocyclization, particularly the retardation of oligomer‐oligomer coupling. The versatility of the approach is further demonstrated through kinetic analysis of existing yield datasets reported in Wilhelmy's classic 1850 paper on chemical kinetics. This approach is practical enough to be integrated into synthetic studies as an add‐on module that provides mechanistic and kinetic insights.

## Graphical abstract

A method to derive kinetic parameters from synthetic studies was developed and named concentration‐dependent yield analysis (CYAN). Using numerical Euler integration for kinetic regression, CYAN was proven adaptable to chemical and operational considerations and revealed unexpected template effects of a transition metal that mediated competitive oligomerization and cyclization via coupling.

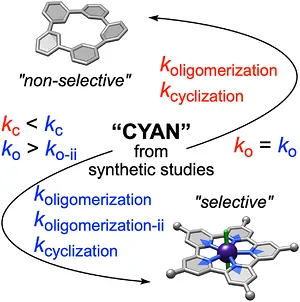

## 1 Introduction

**FIGURE 1:**
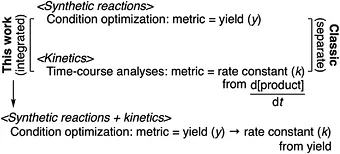
Chemical kinetics and synthetic studies.

One of the most important and practical metrics in synthetic studies is product yield, and chemists strive to optimize reaction conditions to maximize the synthetic efficiency of reactions (Figure 1) [1]. Fundamental features of synthetic reactions, on the other hand, are revealed by kinetic studies through other quantitative metrics such as rate constants [2, 3]. Although both synthesis and kinetics are important for the development of chemistry, separate sets of experiments are usually necessary because kinetic studies require, in general, time‐course analyses [4, 5]. This study shows that in‐depth analyses of yields obtained from optimization processes in synthetic studies can reveal the kinetics of synthetic reactions. In particular, we found that yield analysis for kinetics [6], combined with Euler numerical integration [7], was adaptable enough to allow not only for remodeling kinetic rate equations but also for utilizing existing yield data reported in the synthetic chemistry literature. Our results demonstrate that, with the aid of machine‐learning (ML) augmentation of yield data [8, 9], synthetic studies can be readily integrated with kinetic analysis. This method is simple enough to be incorporated into synthetic studies as an add‐on module that provides mechanistic and kinetic insights in general.

**FIGURE 2:**
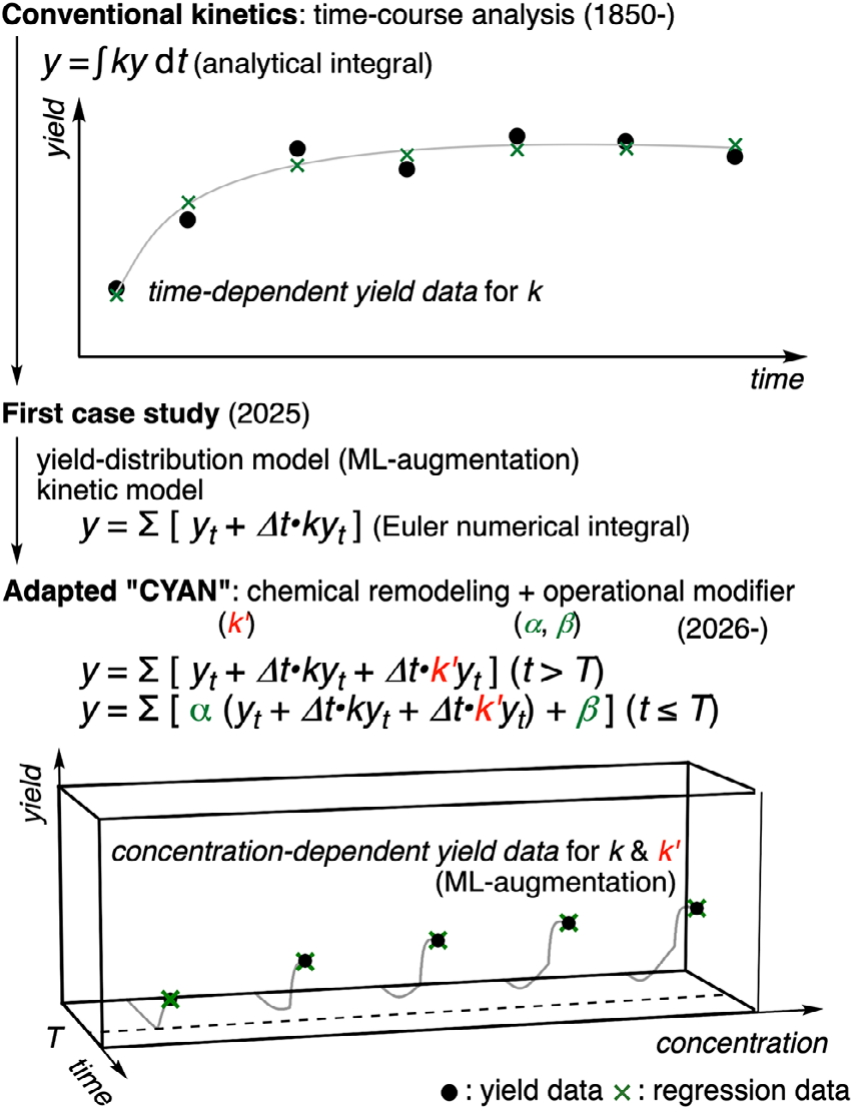
Comparison between conventional time‐course kinetic analysis and the present ML+CYAN approach.

In this study, we integrated chemical kinetics into synthetic studies by taking advantage of modern computational firepower and the science of model building and validation. Importantly, this approach does not rely on conventional time‐course kinetic analysis but instead utilizes two types of models (Figure 2) [10, 11]. The first model is an ML‐based yield‐distribution model (*y*‐model) that augments experimental yield data [9]. The second model is a kinetic model (*k*‐model) that derives rate constants (*k*) from yield data. A prototype protocol for deriving the *k*‐model was originally conceived in our previous study for a specific reaction [6] but was initially found inapplicable to other cases in this study. However, through careful examinations of kinetic rate equations described in the Euler‐integration form, we found the protocol sufficiently versatile and adaptable to merit the name concentration‐dependent yield analysis (CYAN). Importantly, we found that the kinetic rate equations used in CYAN were adaptable to chemical remodeling and operational modification, which enabled us to determine rate constants of various reactions. With the aid of the adapted CYAN protocol, we revealed an important yet unexpected role of a metal template in macrocyclization. Moreover, the combination of ML‐augmentation and CYAN (ML+CYAN) allowed us to derive a reasonable rate constant for acid‐catalyzed hydrolysis of sucrose using existing yield data from a paper published in 1850 [4].

## 2 Results and Discussion

### 2.1 Yield‐Distribution Models for Metal‐Templated Oligomeric Macrocyclization via Coupling

We originally examined the ML+CYAN combination for one reaction [6], but in the present study, investigations of a metal‐templated macrocyclization as the second example revealed that the approach was not directly applicable to other cases. Importantly, however, the protocol was subsequently found to be highly adaptable through chemical remodeling and operational modification, enabling application to diverse reactions.

**FIGURE 3:**
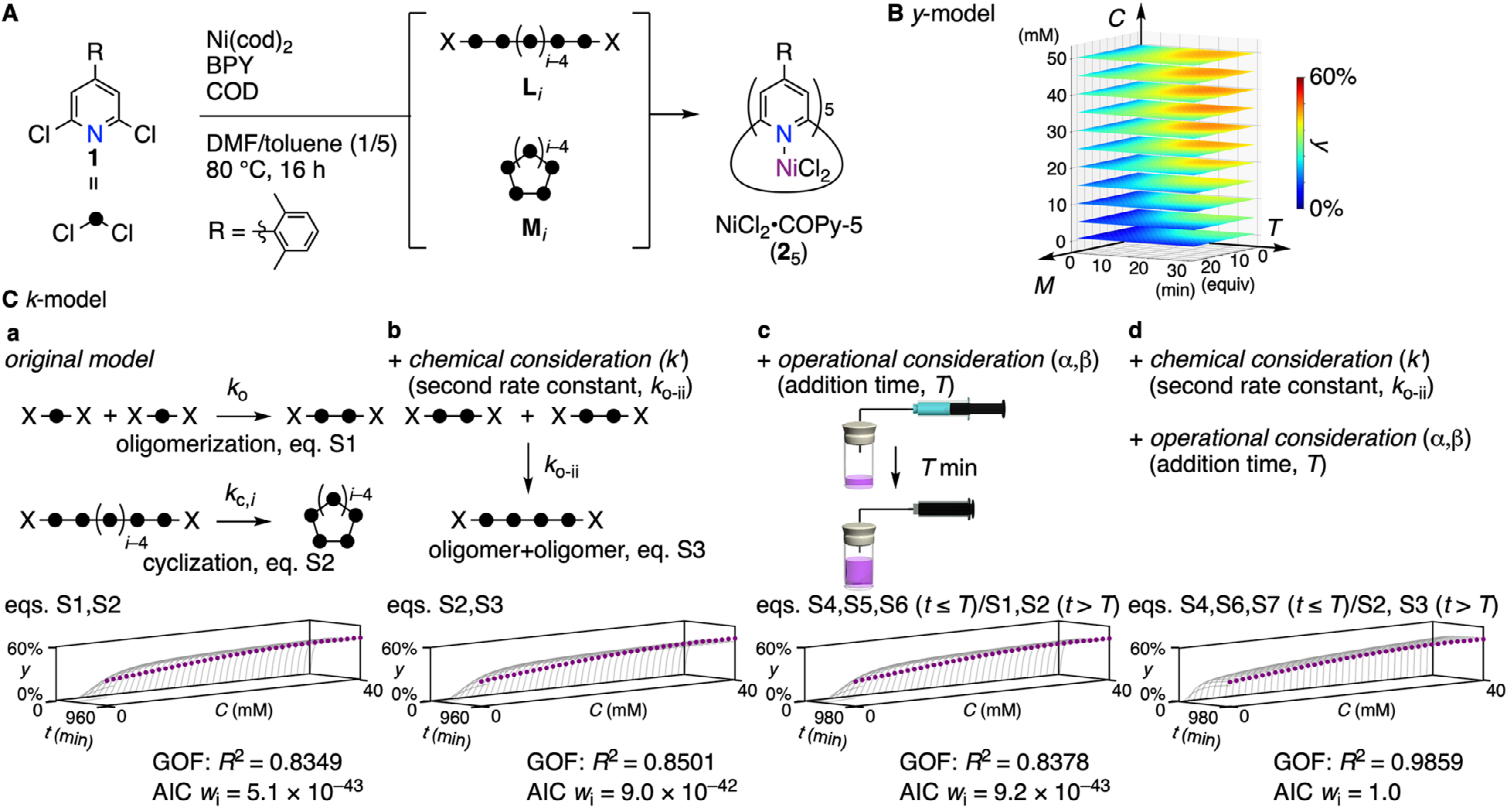
Integration of chemical kinetics into synthetic studies. (A) Synthesis of COPy‐5 (**2**_5_). (B) Yield distribution model (*y*‐model) in a three‐parameter space for MOMC. *M*: equivalents of Ni, *T*: addition time of **1**, *C*: final concentration of **1**. (C) Four different kinetic models (*k*‐models) for CYAN. Conceptual schemes of the kinetic rate equations as well as the regression curves from CYAN are shown. In CYAN graphs, the Euler‐integration curves (gray) are shown along with dots (purple) representing ML‐augmented yield data for **2**_5_.

We first describe the construction of the *y*‐model. We studied the large‐scale synthesis of NiCl_2_‐coordinated pentameric cyclic oligomer of pyridine (NiCl_2_•COPy‐5, **2**_5_) and attempted optimization of the reaction conditions for its synthesis through metal‐templated oligomeric macrocyclization via coupling (MOMC) (Figure 3A) [12]. For the optimization, we examined three factors (*M*,*T*,*C*), where *M* is the equivalents of the Ni reagent, *T* is the substrate addition time and *C* is the final substrate concentration. By performing triplicate reactions at 9 coordinates designated by Taguchi's orthogonal array in the 3D parameter space [13], we obtained 27 datasets of yields (Table S1). The experimental yield datasets were then used to build a *y*‐model by ML analysis using our in‐house code (Supporting Information) [9], affording heatmap models of the yield distribution (Figure S1). Based on the mean‐squared errors (MSEs) and mean absolute percentage errors (MAPEs) of the *y*‐models, the support vector regression (SVR) model was found to be superior to the other three ML methods (Figure 3B and Figure S1).

### 2.2 Kinetic Models for Metal‐Templated Oligomeric Macrocyclization via Coupling

While attempting to construct *k*‐models from yield datasets generated by the *y*‐model of MOMC, we initially noticed that the CYAN process in its original form was not directly applicable to this case. Yield datasets at coordinates of (*M*,*T*,*C*) = (4,20,*C*) were first augmented over a *C* range of 1–40 mM with 1‐mM intervals using the SVR *y*‐model (Table S2). We then employed kinetic rate equations describing the concentrations of linear oligomers ([**L** *_i_*]*_t_*_+_ *_Δt_*) and macrocycles ([**M** *_i_*]*_t_*_+_ *_Δt_*), formulated using Euler numerical integration, and performed least‐squares regression to derive the rate constants (*cf*. Figure S2). In brief, the Equation S1 describes how the concentration of **L** *_i_* increases from [**L** *_i_*]*_t_* through coupling between **L** *_j_* and **L** *_i‐j_* with an oligomerization rate constant *k*_o_, while decreasing through consumption to form longer oligomers (oligomerization rate constant: *k*_o_) and cyclized product **M** *_i_* (cyclization rate constants: *k*_c,_ *_i_*). In contrast, the Equation S2 describes how the concentration of macrocycle **M** *_i_* increases through cyclization of **L** *_i_* with *k*_c,_ *_i_* as the cyclization rate constant. Using these two equations to reproduce 40 yield datasets generated by ML augmentation, we carried out least‐squares regression to determine *k*_o_ and *k*_c,_ *_i_* with our in‐house Python codes and Excel files developed for this study (Supporting Information). Although the regression in our original study converged with a reasonable goodness‐of‐fit (GOF) (*R* ^2^ = 0.9491) [6], we obtained a poorer *R* ^2^ value of 0.8349 by applying the identical process to the MOMC reaction (*k*‐model **a** in Figure 3C).

Examining the Euler‐integration form of the kinetic equations, we noticed that the descriptions were highly amenable to modification and adaptable for incorporating chemical and operational considerations. Two types of modifications were thus examined to make the CYAN process adaptable for constructing various types of *k*‐models (*cf*. Figures S3 and S4). The first modification addressed the chemical differences between templated and non‐templated macrocyclization. Because pyridine oligomers possess chelating ability, oligomeric intermediates during the reaction were expected to alter the oligomerization kinetics [14]. We therefore introduced a second rate constant to describe the coupling between oligomers (*k*' in Figure 2; *k*_o‐ii_ in Figure S3 and Equation S3), separately from the monomer‐based rate constant (*k*_o_) for oligomerization. Although this revised *k*‐model improved the regression, the *R* ^2^ value remained unsatisfactory (*R* ^2^ = 0.8501; **b** in Figure 3C).

We next noticed that the Euler‐formulated rate equations were also highly adaptable for incorporating synthetic operations. The second modification thus addressed operational effects in the rate equations. The MOMC reaction involved dropwise substrate addition as an important synthetic operation, which was included in the optimization coordinates (*M*,*T*,*C*) as *T*. We noticed that the Euler‐formulated rate equations were capable of accommodating relevant modifiers (α and β in Figure 2) to refine the *k*‐model [7]. Thus, during the addition time (*t* ≤ *T*), the addition of the monomeric substrate resulted in dilution of the solution (α modifier) and also in an increase in the concentration of the monomer (β modifier; Figure 2), and the rate equations were modified as Equations S4–S6. After the completion of the substrate addition (*t* > *T*), the kinetics reverted to Equations S1 and S2 without modifiers. Although this model (**c** in Figure 3C) did not dramatically improve the regression, the combined model incorporating the second rate constant, *k*_o‐ii_, as well as the α and β modifiers resulted in considerable improvement, giving an *R* ^2^ value of 0.9859 (**d** in Figure 3C). As the models were mathematically nested [10, 15], model comparison was performed using Akaike's information criterion (AIC) (Table S3) [16]. The AIC weight, *w*_i_, representing the weight of evidence, strongly supported the model **d** as the optimal model (AIC *w*_i_ = 1.0) from an information‐theoretic perspective [10, 17].

### 2.3 Template Effects in Metal‐Templated Oligomeric Macrocyclization via Coupling

**FIGURE 4:**
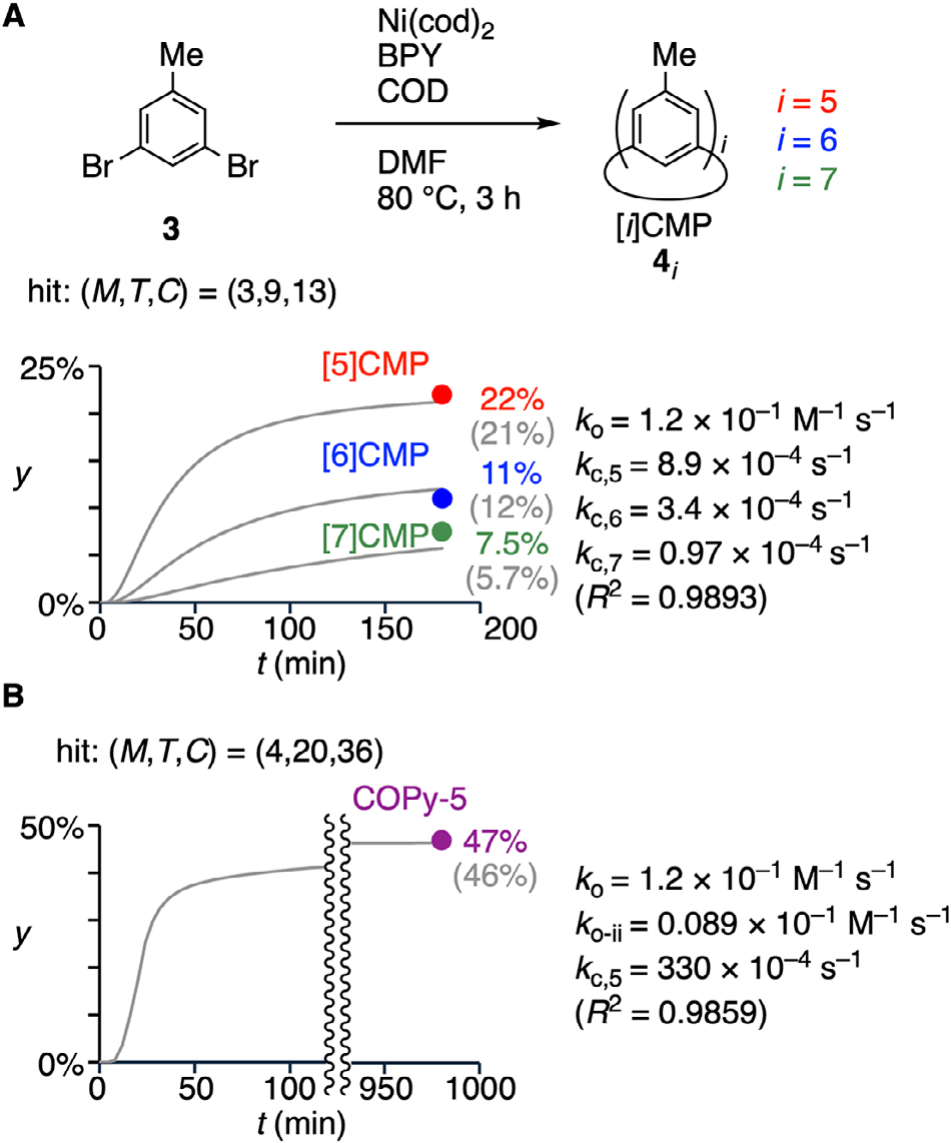
Kinetic features of non‐templated and templated macrocyclization via coupling. (A) A reference macrocyclization for the synthesis of [*i*]CMP (**4** *_i_*). The kinetic Euler‐integration curves (gray) at the hit coordinate are shown with the experimental yield data as dots. (B) The kinetic Euler‐integration curve (gray) at the hit coordinate for the synthesis of COPy‐5 (**2**_5_) is shown with the experimental yield data as a dot.

Additional investigations of a non‐templated macrocyclization reaction allowed us to reveal details of the template effects in the MOMC reaction [18, 19]. We thus performed 27 reactions for the synthesis of *i*‐mer cyclo‐*meta*‐phenylenes ([*i*]CMP, **4** *_i_*) as a reference (Figure 4A) and characterized the kinetics of the non‐templated reaction using the ML+CYAN approach. Following the procedure used for MOMC, we built the SVR *y*‐model (Figures S5 and S6 and Tables S4 and S5) as well as the *k*‐model for the non‐templated oligomeric macrocyclization of **3** to **4** *_i_* (Figure S7). The CYAN analysis of the non‐templated reactions converged and resulted in an excellent *R* ^2^ value of 0.9893 with the *k*‐model **a**. The kinetic curves obtained by Euler integration using the rate constants for oligomerization (*k*_o_) and macrocyclization (*k*_c,5_, *k*_c,6_ and *k*_c,7_) are shown for the hit coordinate (*M*,*T*,*C*) = (3,9,13) in Figure 4A. Performing a test run of the reaction at this hit coordinate, we recorded the yields of [5]‐, [6]‐ and [7]CMPs as 22%, 11% and 7.5%, which were in good agreement with the simulated values (21%, 12% and 5.7%) obtained with the CYAN rate constants. In addition, the *k*_o_ value derived from CYAN reasonably agreed with the kinetic data reported by Yamamoto (see below) [14]. The validity of the models for MOMC was likewise confirmed by performing an additional test run reaction at the hit coordinate. The experimental yield of 47% at (*M*,*T*,*C*) = (4,20,36) was reproduced as 46% by the kinetic simulation performed with the CYAN rate constants, as shown in Figure 4B with the kinetic Euler curve and the experimental yield data.

As the models have been validated by experiments, we discuss and summarize the kinetic features of the Ni template in MOMC. First, the Ni template accelerated the cyclization process. The cyclization rate constant for [5]CMP (*k*_c,5_) was 8.9 × 10^−4^ s^−1^, which dramatically increased to 330 × 10^−4^ s^−1^ for COPy‐5 in the templated reaction. Second, an unexpected role of the Ni template in retarding reactions was revealed. In MOMC, the oligomer‐oligomer coupling was found to be dictated by a different rate constant, *k*_o‐ii_, which was, importantly, an order of magnitude smaller than that of the monomer‐involving oligomerization (*k*_o‐ii_ = 0.089 × 10^−1^ m ^−1^ s^−1^ and *k*_o_ = 1.2 × 10^−1^ M^−1^ s^−1^) [14]. The rate constant for oligomer‐oligomer coupling thus dropped to 7% of that for monomer‐involving oligomerization. We believe that, capturing Ni centers, multidentate oligopyridines become sterically hindered to retard important elementary steps such as disproportionation [14]. Consequently, during the MOMC reaction, the oligomers are extended in a stepwise manner to increase the length one by one, and upon reaching the cyclizable length, the macrocycle is rapidly formed. Through these kinetic effects, COPy‐5 can be selectively obtained as the major product, unlike the non‐templated [*i*]CMP synthesis, which inevitably affords macrocycles as a mixture of various ring sizes.

### 2.4 Versatility of ML+CYAN Approach

**FIGURE 5:**
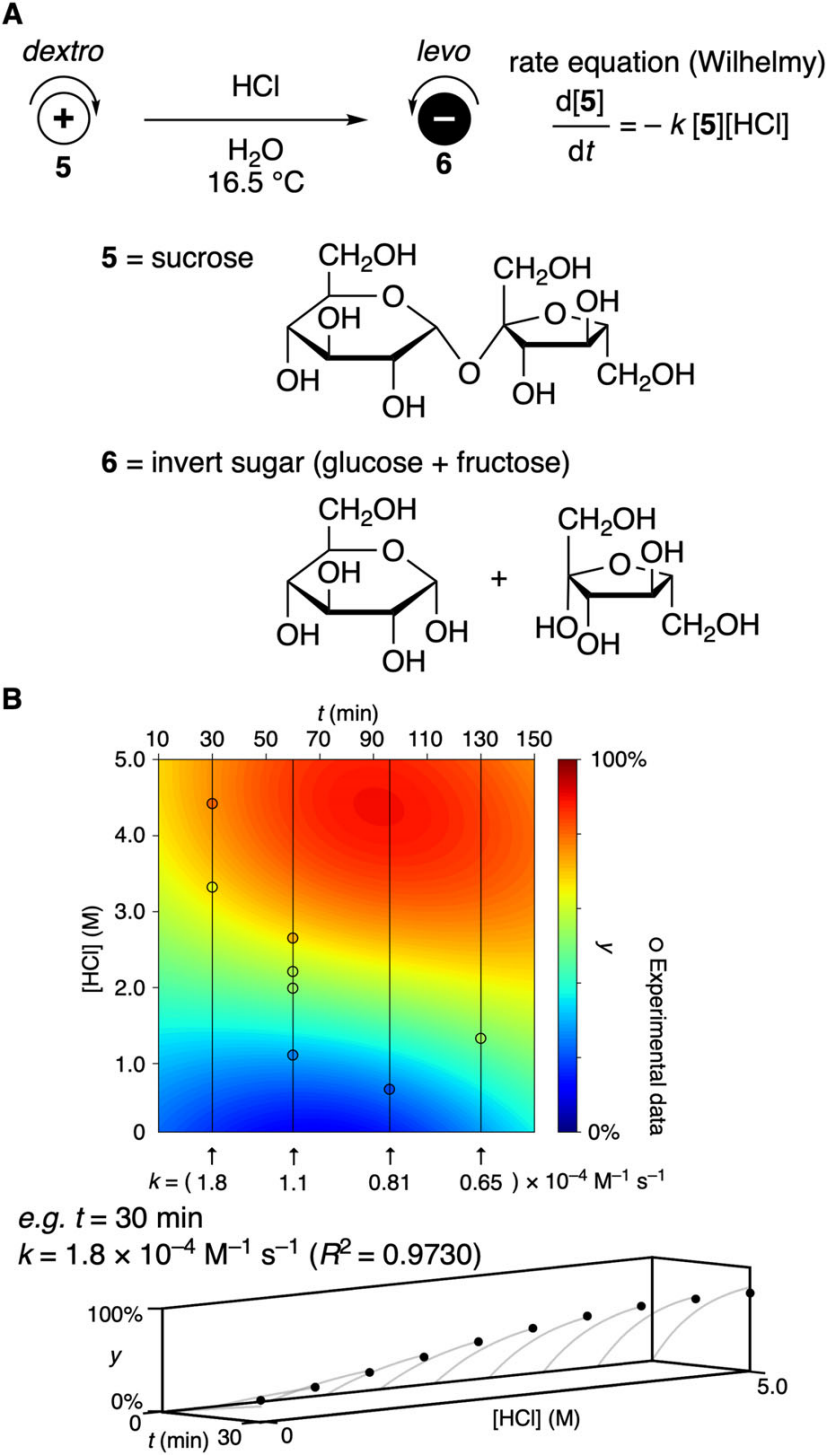
Revisiting the origin of chemical kinetics with the ML+CYAN approach to reveal the rate constants from existing datasets. (A) Synthesis of invert sugar. The kinetic equation introduced by Wilhelmy in 1850 is reformulated in modern notation with the following substitutions: *k* = *M*, [**5**] = *Z* and [HCl] = *S* [4]. (B) Two models (*y*‐model and *k*‐model) for invert sugar synthesis by Wilhelmy. For the *y*‐model, *t* = reaction time and [HCl] = concentration of HCl. Data reported in Wilhelmy's paper are shown as dots. For the *k*‐model, the Euler‐integration curves (gray) are shown together with yield datasets as dots (black), with *t* = 30 min as an example. See also Figure S9.

Finally, we wish to exemplify the broad utility of the ML+CYAN approach. The CYAN process is applicable to any type of concentration‐dependent yield data, provided the yield depends on the concentration of a substrate or reagent (**x**), i.e., *y* = *f*([**x**]). In addition, the ML augmentation process is useful for expanding concentration‐dependent yield datasets not only in ongoing optimization studies but also in existing datasets, which should even allow reexamination of the kinetics behind published yield data. In this study, we decided to reexamine the data reported in 1850, revisiting the very first paper on chemical kinetics by Wilhelmy [4]. In Wilhelmy's paper, the kinetic rate equation was introduced for the first time to quantitatively characterize chemical reactions (Figure 5A) [20], and Wilhelmy studied the conversion of sucrose (dextrorotatory sugar) to a so‐called invert sugar (levorotatory sugar) [21], intriguingly, without knowing the structures of the compounds [22]. The progress of the reaction was monitored over the course of the reaction using optical rotation data to quantify the reactant (sucrose, **5**) and the product (invert sugar, **6**), and the rate constant *k* was determined by using the equation for the bimolecular reaction described in d[**5**]/d*t* = –*k*[**5**][HCl] (*k* was abbreviated as *M* in his study). Since this pioneering report, time‐course analysis has been widely utilized to reveal reaction kinetics [2, 3].

Wilhelmy supplemented concentration‐dependent yield data in his accompanying paper during his optimization studies [4, 23], and among various datasets of Wilhelmy's investigations, we found an appropriate table to be subjected to the ML+CYAN analysis. Thus, [HCl]‐dependent yield datasets were recorded in section I of Table V (Figure S8 and Table S6; 8 datasets) [23], and with these datasets, we first constructed an SVR *y*‐model. As shown in a 2D heatmap (Figure 5B), the *y*‐model augmented concentration‐dependent yield datasets in a parameter space composed of reaction time, *t*, and concentration of hydrochloric acid, [HCl]. We then extracted 20 [HCl]‐dependent yield datasets from the *y*‐model at *t* = 30, 60, 96 and 130 min (Tables S7–S10) and determined the rate constant (*k*) at each reaction time by CYAN. Albeit minute deviations with the reaction time, the rate constants were consistent with each other and fell within the range of *k* = (1−2) × 10^−4^ M^−1^ s^−1^ (Figure 5B and Figure S9). As a possible source of the deviations, Wilhelmy himself noted in his first paper [4]: “The small fluctuations in the value of *M* correspond to the temperature (Die geringen Schwankungen im Werthe von *M* entsprechen der Temperatur).”

The HCl‐catalyzed synthesis of invert sugar from sucrose was reexamined in 2007 by time‐course analysis [24], and the data provided in the modern study confirmed the credibility of the CYAN‐derived rate constants. In this modern analysis, the rate constant was recorded as *k*' = 1.8 × 10^−5^ s^−1^ by approximating the reaction with the pseudo‐first‐order equation d[**5**]/d*t* = −*k*'[**5**]. The approximated *k*' value can then be converted to the actual bimolecular rate constant, *k*, using the reported condition of “[HCl] = 0.1 N” to give *k* = 1.8 × 10^−4^ M^−1^ s^−1^. Considering variations in the HCl concentration of reagent‐grade chemicals, we conclude that the CYAN‐derived rate constants (Figure 5B and Figure S9) matched nicely with the modern kinetic data from the time‐course investigations.

## 3 Conclusion

In this study, we demonstrated the versatility of the ML+CYAN approach to reveal the fundamental kinetics behind synthetic yield data without recourse to separate, elaborate kinetic experiments [25]. Unexpectedly, the Euler integration implemented in the CYAN process was found to be adaptable to incorporate chemical and operational considerations, thereby providing an unconventional yet powerful framework for kinetic analysis based on synthetic yield distributions. The ML+CYAN approach succeeded not only in locating the optimal condition in the parameter space for the synthesis but also in revealing previously unrecognized mechanistic roles of a transition‐metal template in coupling‐mediated oligomeric macrocyclization. Thus, comparison with a reference non‐templated macrocyclization revealed that, expectedly, cyclization was accelerated and, unexpectedly, oligomer‐oligomer coupling was retarded by the Ni template. Such kinetic retardation may be a common feature in transition‐metal‐mediated oligomerization of coordinating substrates and may provide mechanistic insight into related reactions in general [26]. The Ni‐mediated coupling studied here is known as the Yamamoto coupling and has been widely adopted in modern synthetic studies of nanocarbon molecules [27, 28]. The kinetic features revealed in this study should thus provide valuable mechanistic insights for future studies. In addition, the applicability of the ML+CYAN approach was further demonstrated through extraction of a reasonable rate constant from Wilhelmy's concentration‐dependent yield data reported in the very first paper on chemical kinetics published in 1850 [4]. We believe that the present methodology should provide a broadly applicable framework for integrating kinetic analysis into synthetic studies using concentration‐dependent yield datasets. Although the prototype approach originally emerged as a “lucky guess” [6, 11], the present study demonstrates that CYAN is highly adaptable to accommodate various mechanistic and synthetic considerations, with the ML+CYAN approach proving useful for reanalysis of existing synthetic datasets. As was the case for the classic E1/E2 concept [2, 29, 30], an initial “lucky guess” cannot become a useful methodology until its generality is demonstrated and the concept is established with an appropriate name [31]. All the in‐house Python codes are provided, and we hope that the readers will examine the applicability to many other cases to deepen understanding of chemical reactions through synthetic studies. Note that concentration‐dependent yield data obtained, for instance, at three concentrations in conventional synthetic optimization can also be subjected to CYAN to provide quantitative kinetic measures in addition to the identification of optimized conditions. In addition, for the present study, we limited our investigation to the interpolation regime of the experimental parameter space. Extrapolation with ML methods or expansion of the experimental parameter space with additional experiments may be exploited for reaction optimization and kinetic analysis over a wider concentration range [32]. Furthermore, we hope that this study may promote further interplay between ML/AI and experimental synthetic studies, together with advances in ML/AI‐driven exploration of chemical kinetics [33].

## Author Contributions

**Xinyi Xiao**: investigation, methodology, visualization, formal analysis, validation, writing – original draft, writing – review and editing. **Koki Ikemoto**: methodology, investigation, visualization, writing – review and editing, formal analysis, validation, funding acquisition. **Toshiya M. Fukunaga**: writing – review and editing, formal analysis, validation, funding acquisition. **Hideaki Nishina**: investigation, writing – review and editing, formal analysis. **Hiroyuki Isobe**: conceptualization, writing – original draft, writing – review and editing, supervision, visualization, funding acquisition.

## Conflicts of Interest

The authors declare no conflicts of interest.

## Supporting information




**Supporting File 1**: advs77694‐sup‐0001‐SuppMat.pdf.

**Supporting File 2**: advs77694‐sup‐0002‐SuppMat.zip.

## Data Availability

The data that supports the findings of this study are available in the supplementary material of this article.
