# Supplementary material for: Integrating Kinetics into Synthetic Studies via Adaptable Concentration‐Dependent Yield Analysis
Source: Adv Sci (Weinh). 2026 Sep 27:e77694. Online ahead of print. doi: 10.1002/advs.77694 (PMC13616260; doi:10.1002/advs.77694)
Supplement: Supplementary file 1 [file ADVS-9999-e77694-s001.pdf]

Supporting Information

Table of contents

Supplementary Methods 2

Supplementary Figures 8

Supplementary Tables 13

Supplementary Equations 16

Supplementary Codes 18

Supplementary References 28

Supplementary Methods

Materials

Anhydrous DMF (Kanto), and toluene (Kanto) were purified by a solvent purification system (GlassContour) equipped with columns of activated alumina and supported copper catalyst (Q-5) [34]. Dichloropyridine **1** was prepared by the methods reported in the literature [12]. All other chemicals were of reagent grade and used without any further purification.

Yield dataset for $NiCl_2$•COPy-5

Yield datasets for $NiCl_2$•COPy-5 (**2**$_5$) were obtained from synthetic experiments. We performed triplicate reactions with **1** under nine different conditions designated by (M,T,C) coordinates in the parameter space. The coordinates were set according to Taguchi's $L_9(3^4)$ array, namely, (5,1,1), (5,5,10), (5,20,40), (10,5,1), (10,20,10), (10,1,40), (20,20,1), (20,1,10) and (20,5,40) [13]. Taking (M,T,C) = (10,5,1) as a typical example, the synthetic procedure is described. A mixture of 2,2'-bipyridine (BPY; 156 mg, 1.00 mmol), 1,5-cyclooctadiene (COD; 0.123 mL, 1.00 mmol) and bis(1,5-cyclooctadiene)nickel(0) ($Ni(cod)_2$; 274 mg, 1.00 mmol) in DMF (17.0 mL) and toluene (17.0 mL) was stirred at 80 °C for 30 min. To the mixture was added a solution of **1** (25.2 mg, 0.100 mmol) in toluene (66.5 mL) dropwise over 5 min, and the mixture was stirred at 80 °C. After 960 min, 2 M aq. HCl (2.5 mL) was added, and the mixture was vigorously stirred overnight. The mixture was diluted with chloroform (20 mL), and after phase separation, the aqueous layer was extracted with chloroform (20 mL × 2). The combined organic layers were washed with brine, dried over Na_2SO_4 and concentrated in vacuo. Analysis of the crude material by MALDI mass spectrometry (matrix = dithranol, Bruker Daltonics Autoflex speed) showed the presence of minor products, with the 8-mer oligomer as the largest detectable compound. The crude material was dissolved in $CDCl_3$, and bromoform (12.2 mg, 48.3 μmol) was added as an internal standard. The relaxation delay was extended to 30 s for quantitative measurement, and the ^{1}H NMR spectrum was recorded (JEOL RESONANCE JNM-ECAII 600). Using a doublet resonance of $NiCl_2$•COPy-5 (**2**$_5$) at 6.36 ppm as the reference, the amount of $NiCl_2$•COPy-5 (**2**$_5$) was determined to be 2.08 μmol (yield = 10%). Triplicate reactions were performed at each (M,T,C) coordinate, and we obtained 27 yield datasets in total (Table S1).

Yield-distribution model (y-model) for $NiCl_2$•COPy-5

Yield-distribution models (y-models) for $NiCl_2$•COPy-5 were constructed by using the yield datasets in Table S1 with four ML methods, namely, support vector regression (SVR), partial least squares regression (PLSR), multilayer perceptron (MLP) and random forest (RF) [9]. The procedure adopting libraries of NumPy (v. 2.3.2) [35], pandas (v. 2.3.1) [36], matplotlib (v. 3.10.5) [37] and scikit-learn (v. 1.7.1) [38] in Python (v. 3.12.2) is briefly described. The initial grid range for ML hyperparameters were $1 \times 1 \leq C \leq 1 \times 50$, $0.1 \times 1 \leq \varepsilon \leq 0.1 \times 20$ and $0.1 \times 1 \leq \gamma \leq 0.1 \times 20$ for SVR, 1 ≤ n-components

≤ 10 and 1 ≤ degree ≤ 10 for PLSR, hidden layer size = 10, 50, 100, 500 and 1000 for MLP and 1 × 1 ≤ max depth ≤ 1 × 9 for RF. A representative code for grid search with SVR is provided as Code S1. Using the leave-one-out cross-validation method [39], hyperparameters were optimized ($C = 49$, $\varepsilon = 1.3$, $\gamma = 0.2$ for SVR; n-components = 8, poly degree = 2 for PLSR; hidden layer size = 500 for MLP; max depth = 8), and upon regression convergence, the y-models in the form of heatmaps were constructed using Code S2. As shown in Figure S1, ML models were evaluated by the mean-squared errors (MSEs) and mean absolute percentage errors (MAPEs), and the SVR model was found to be superior to the other three models. The SVR y-model showed the highest yield of 47% at the hit coordinate of $(M,T,C) = (4,20,36)$, and the 40×2 concentration-dependent yield datasets for CYAN were extracted at the coordinate of $(4,20,C)$ over the C range of 1 to 40 mM with 1-mM intervals (Table S2). When the hyperparameters for the SVR model were modified to an arbitrary set ($C = 48$, $\varepsilon = 1.2$, $\gamma = 0.1$), the rate constants (k_o, $k_{o\text{-ii}}$ and $k_{c,5}$) in k-model **d** remained unchanged to two significant figures, thereby corroborating the stability of the analysis. For more extensive evaluation of the ML component of the ML+CYAN strategy, additional regression methods, such as Gaussian process regression or gradient boosting, may be examined, together with external test sets or repeated k-fold cross-validation with reported variance [40].

Kinetic model (*k*-model) for $NiCl_2$•COPy-5: Kinetic rate equations

To construct k-models via CYAN, four different sets of kinetic rate equations in the Euler-integration form were developed. The development of the equations based on chemical and synthetic considerations prior to regression analysis is first described. The first model (k-model **a**) is based on the consideration that two types of rate constants are involved in MOMC: one for oligomerization (k_o) and the other for cyclization (Figure S2) [6]. Considering that the rate constants for cyclization vary depending on the ring size, separate rate constants are defined for formation of each i-mer macrocycle ($k_{c,i}$) with the range of i values set by the species detected by MS. Macrocycles smaller than the isolated species were neither detected nor experimentally isolated, making the treatment of $k_{c,n} = 0$ reasonable. Detected macrocycles larger than the isolated species were taken into consideration in the y_R terms, whereas the rate constants for undetected larger macrocycles were reasonably set to zero. This treatment assumes that the undetected species are produced in minute amounts and do not substantially contribute to the yield distributions. Under these considerations, the Euler-formulated rate equation for formation of i-mer linear oligomers $[\mathbf{L}_i]_{t+\Delta t}$ via oligomerization is described in eq. S1, whereas that for formation of i-mer macrocycles $[\mathbf{M}_i]_{t+\Delta t}$ via cyclization is described in eq. S2. The development of the adapted equations is next described. The second model (k-model **b**) is based on chemical considerations at the oligomerization steps (*cf.* k' in Figure 2). When multiple pyridine units are covalently connected in the coupling substrates, multidentate chelation by the nitrogen atoms becomes possible and may affect the subsequent coupling reactions [26]. As

shown in Figure S3, we can therefore introduce a second rate constant at the oligomerization steps to differentiate monomer-based oligomerization (k_o) and oligomer-oligomer coupling ($k_{o\text{-}ii}$). Under these considerations, the Euler-formulated rate equation for formation of i-mer linear oligomers $[\mathbf{L}_i]_{t+\Delta t}$ via oligomerization is reformulated as eq. S3, whereas that for formation of i-mer macrocycles $[\mathbf{M}_i]_{t+\Delta t}$ via cyclization remains unchanged as eq. S2. The third model (k-model **c**) is based on synthetic considerations (*cf.* α and β in Figure 2). In MOMC, the monomeric substrate **1** (= $\mathbf{L}_1$) is introduced over the addition time T, resulting in both dilution of the reaction mixture (α) and increase of the monomer concentration $[\mathbf{L}_1]$ (β). As shown in Figure S4, defining the final volume as $3v$ and reaction time and time increment as t and Δt, respectively, we can introduce two modifiers, α and β, defined as the function of T, v, t and Δt. Under these considerations, during the addition time ($t \leq T$), the Euler-formulated rate equation for monomer concentration $[\mathbf{L}_1]_{t+\Delta t}$ and for formation of i-mer linear oligomers $[\mathbf{L}_i]_{t+\Delta t}$ via oligomerization is reformulated as eqs. S4 and S5, whereas that for formation of i-mer macrocycles $[\mathbf{M}_i]_{t+\Delta t}$ via cyclization is reformulated as eq. S6. After the addition time ($t > T$), the equations return to original forms described in eqs. S1 and S2. The fourth model (k-model **d**) is based on a combination of the chemical and synthetic considerations. Under these considerations, during the addition time ($t \leq T$), the Euler-formulated rate equation for formation of i-mer linear oligomers $[\mathbf{L}_i]_{t+\Delta t}$ via oligomerization is reformulated as eq. S7, whereas those for $[\mathbf{L}_1]_{t+\Delta t}$ and $[\mathbf{M}_i]_{t+\Delta t}$ remain unchanged from eqs. S4 and S6, respectively. After the addition time ($t > T$), the equations return to eqs. S3 and S2.

Kinetic model (k-model) for $NiCl_2$•COPy-5: Regression and validation

Kinetic models (k-models) for $NiCl_2$•COPy-5 were constructed from the concentration-dependent yield datasets in Table S2 by using eqs. S1-S7 to derive kinetic rate constants via regression. For the CYAN regression, we developed Excel files and Python codes using libraries of NumPy (v. 2.4.2) [35], xlwings (v. 0.33.15) [41] and SciPy (v. 1.17.0) [42] in Python (v. 3.13.7). Each Excel file performs the Euler integrations based on the kinetic rate equations, and Python performs least-squares regression to optimize the rate constants. Four Excel files were prepared for the numerical Euler integration (Data S1-S4). As an example, the procedure for the first k-model **a** is described. The Excel file, Data S1, comprised two types of spreadsheets: one for regression ("CYAN regression" tag) and others for integration ("Euler" tags). The regression spreadsheet has two roles. First, it allows us to input initial values of the rate constants (k_o, $k_{c,5}$, $k_{c,6}$, $k_{c,7}$, $k_{c,8}$; red fonts) and ML-based 40×2 yield datasets (y_5 for $\mathbf{2}_5$ and y_R for others; blue fonts). Second, it allows the Python program to refer to cells enclosed by bold borders. The Euler-integration spreadsheets comprise 40 sheets corresponding to the C (= $[\mathbf{L}_1]_0$) range of 1-40 mM with 1-mM intervals. Each Euler spreadsheet performs numerical Euler integration for $[\mathbf{L}_i]_{t+\Delta t}$ and $[\mathbf{M}_i]_{t+\Delta t}$ with $\Delta t = 1$ by using eqs. S1 and S2, respectively, under designated $[\mathbf{L}_1]_0$ (orange background). Within the Euler spreadsheet, the concentrations at $t = 960$ are

then converted into yield datasets (y_5, y_{R_Mi} and y_{R_Li}), which are returned to the regression spreadsheets (yellow background). In addition, the smallest yield value (y_{MIN}) and the largest yield value (y_{MAX}) within the Euler spreadsheet are summarized (yellow background), and the smallest and the largest values among all the Euler spreadsheets are returned to the regression spreadsheets. Using the returned values, the regression spreadsheet calculates squared residuals and squared deviations from the mean (green background) by comparison of the yield datasets. These values are then used to calculate the sum of squared residuals, sum of squares total and R^2 values (green background). The Python program directed by Code S3 then reads the value written in the cell corresponding to the sum of squared residuals (bold-bordered) and performs least-squares regression to optimize the rate constant values with constraints requiring $y_{MIN} \geq 0$ and $y_{MAX} \leq 100$ for the minimum and maximum yield values (bold-bordered). The constrained optimization is performed using the sequential least squares programming (SLSQP) algorithm implemented in SciPy, and after each optimization cycle, Python overwrites the rate constants in the regression spreadsheet (purple background) in the Excel file via xlwings. The optimization process is repeated until convergence is achieved under the condition of changes in sum of squares residuals ≤0.00001. The appropriateness of a *k*-model can be examined using the R^2 value as a measure, and the model should be reconsidered when the R^2 value is insufficient. For remodeling, the following combinations were adopted: *k*-model **b** = Data S2 and Code S3 (with minor changes for file and rate constant designation); the *k*-model **c** = Data S3 and Code S3 (with minor changes for file and rate constant designation); *k*-model **d** = Data S4 and Code S3 (with minor changes for file and rate constant designation). Although we did not encounter instability or error-propagation issues with Euler integration in the present case, implementation of higher-order Runge–Kutta methods may be useful for more complicated systems [43]. Among the four *k*-models, the *k*-model **d** afforded the highest R^2 value (0.9859) and was validated by an additional experimental reaction under conditions of (M,T,C) = (4,20,36) (experimental yield = 47% and *k*-model **d** = 46%). Notably, the same test-run experiment also validated the *y*-model, with the experimental yield (47%) matching the predicted yield (47%). Comparisons of four *k*-models were performed by using Akaike's information criterion (AIC) (Table S3) [16,17]. The kinetic curves in Figure 3 were drawn using Code S4 (with a minor modification for *k*-model **c** and **d**), whereas the curves in Figure 4 were drawn using Excel.

Reference investigations with [*i*]CMP

Reference investigations for non-templated oligomeric macrocyclization were performed for the synthesis of [*i*]CMP (**4**$_i$). Experimental yield datasets were obtained from triplicate reactions with **3** under nine different conditions at (M,T,C) = (2,1,1), (2,5,10), (2,20,40), (5,5,1), (5,20,10), (5,1,40), (10,20,1), (10,1,10) and (10,5,40) according to Taguchi's $L_9(3^4)$ array [13]. The synthetic procedures were essentially identical to those described above for $NiCl_2$•COPy-5 using **3** (50.0 mg, 0.200 mmol)

as the substrate and DMF (7.0 mL + 13.0 mL) as the solvent with a reaction time of 180 min. Analysis of the crude material by MALDI mass spectrometry (matrix = tetracyanoquinodimethane) showed the presence of minor products, with the 10-mer oligomer as the largest detectable macrocycle. Using singlet peaks of [*i*]CMP $\mathbf{4}_i$ (i = 5, 6, 7) at 8.27, 8.13 and 7.81 ppm as the reference [18], the yields of [*i*]CMP were determined (i = 5: 8.3%; i = 6: 14%; i = 7: 6.9%). Triplicate reactions were performed at each (M,T,C), and 27 yield datasets were obtained in total (Table S4). The SVR *y*-model of [5]CMP was found to be superior among the three ML models (Figure S5) based on the MSE values with the hit coordinate at (M,T,C) = (3,9,13). In addition, *y*-models of [6]- and [7]CMP were also constructed and visualized (Figure S6). By applying non-templated rate equations (model **a**) to the concentration-dependent yield datasets of [*i*]CMP in Table S5, we constructed the *k*-model and obtained the rate constants by CYAN at (M,T,C) = (3,9,C) through the same procedure described for $NiCl_2$•COPy-5 using Data S5 and Code S3. The *k*-model was validated by an additional experimental reaction under conditions of (M,T,C) = (3,9,13), showing good agreement between experimental values and values predicted by kinetics (experimental yield: [5]CMP = 22%, [6]CMP = 11% and [7]CMP = 7.5%; *k*-model: [5]CMP = 21%, [6]CMP = 12% and [7]CMP = 5.7%). The kinetic curves in Figure S7 were drawn using Code S4, whereas those in Figure 4 were drawn using Excel.

Synthesis of invert sugar: Reanalysis of 1850 datasets by ML+CYAN

The synthesis of invert sugar by Wilhelmy was reanalyzed by the ML+CYAN approach. We used one set of [HCl]-dependent yield datasets from the Wilhelmy's paper (Figure S8) [23]. The classic physical notation was first converted to modern notation (Table S6). The optical rotation values recorded as "Z_0" corresponded to the initial concentration of **5**, whereas those denoted as "*D*" were the optical rotation values recorded during hydrolysis at reaction time "*T*". Wilhelmy converted "*D*" to "*Z* Beobachtet. (obsd)", which corresponded to the remaining amount of **5**. Thus, under the assumption of quantitative conversion from **5** to **6**, the yield of **6** was calculated in modern notation as $(1 - Z/Z_0) \times 100$. The value denoted as "*S*" represented the weight of HCl and was divided by "*w*", the amount of water, to convert the values to [HCl] in modern notation. A yield-distribution model (*y*-model) for invert sugar (**6**) was constructed using the yield datasets in Table S7 with the SVR method in a two-dimensional parameter space composed of t and [HCl]. Using Code S1 with the initial settings of the grid range for ML hyperparameters of $3 \times 1 \leq C \leq 3 \times 45$, $0.1 \times 1 \leq \varepsilon \leq 0.1 \times 50$ and $0.1 \times 1 \leq \gamma \leq 0.1 \times 20$, the *y*-model shown in Figure 5B were obtained (Code S5 used for heatmap generation). Using the *y*-model, [HCl]-dependent yield dataset were augmented over the [HCl] range of 0.5-5.0 M with 0.5-M intervals at t = 30, 60, 96 and 130 min (Tables S7-S10). To construct a *k*-model via CYAN for invert sugar, two rate equations in the Euler-integration form were developed (eqs. S8 and S9). The CYAN process was essentially identical to that described for $NiCl_2$•COPy-5. Euler integration was performed with Δt = 0.1 at the reaction times reported in Wilhelmy's paper

using the following combinations: *k*-model (t = 30) = Data S6 and Code S3 (with minor changes for file and rate constant designation); *k*-model (t = 60) = Data S7 and Code S3 (with minor changes for file and rate constant designation); *k*-model (t = 96) = Data S8 and Code S3 (with minor changes for file and rate constant designation); *k*-model (t = 130) = Data S9 and Code S3 (with minor changes for file and rate constant designation). The derived *k* values are included in Data S6-S9, and the kinetic curves are shown in Figures 5B and S9.

Supplementary Figures

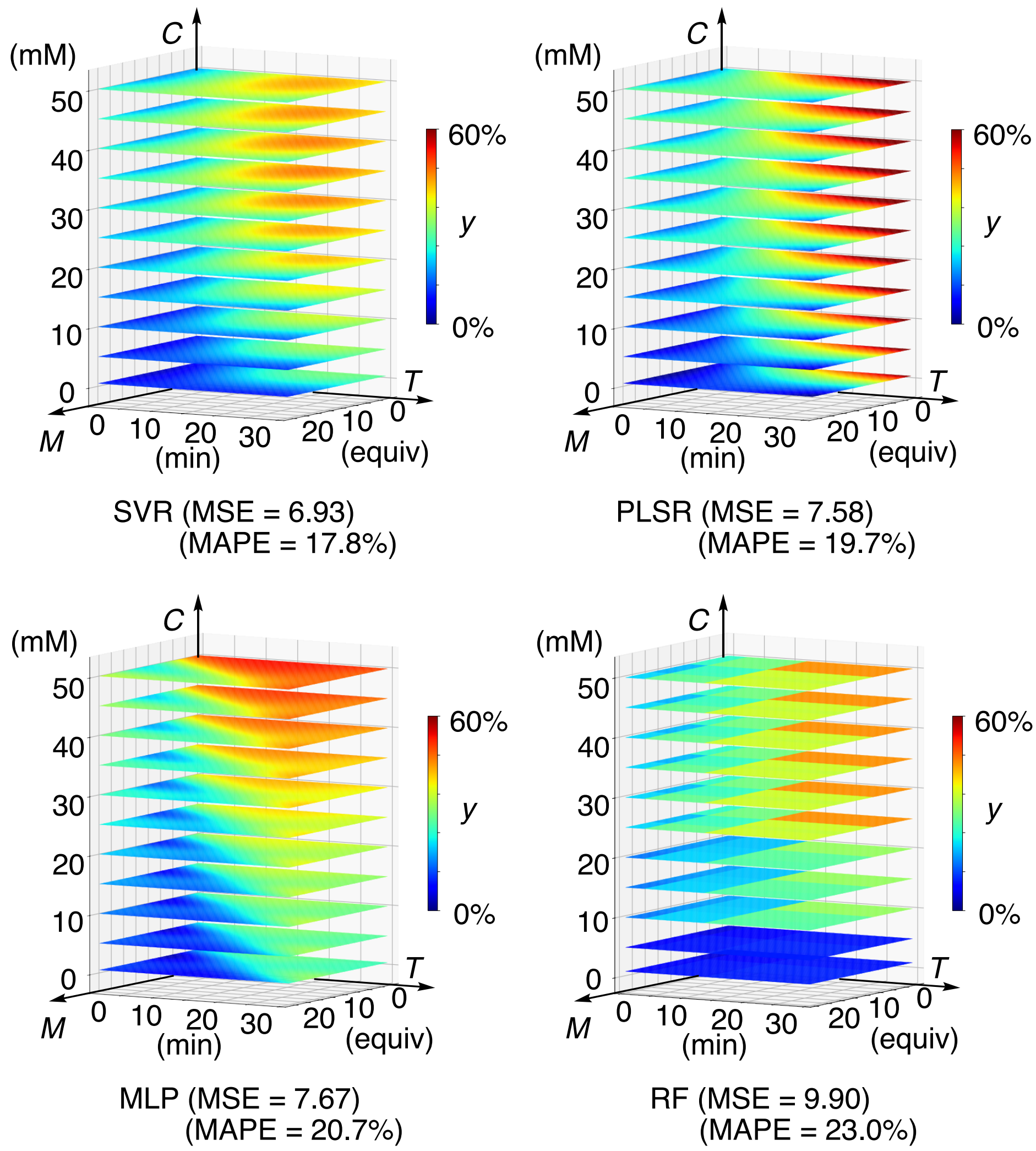

Figure S1. Yield distribution models (y-models) for $\mathbf{2}_5$. The ML methods and MSE and MAPE values are shown under the heatmaps.

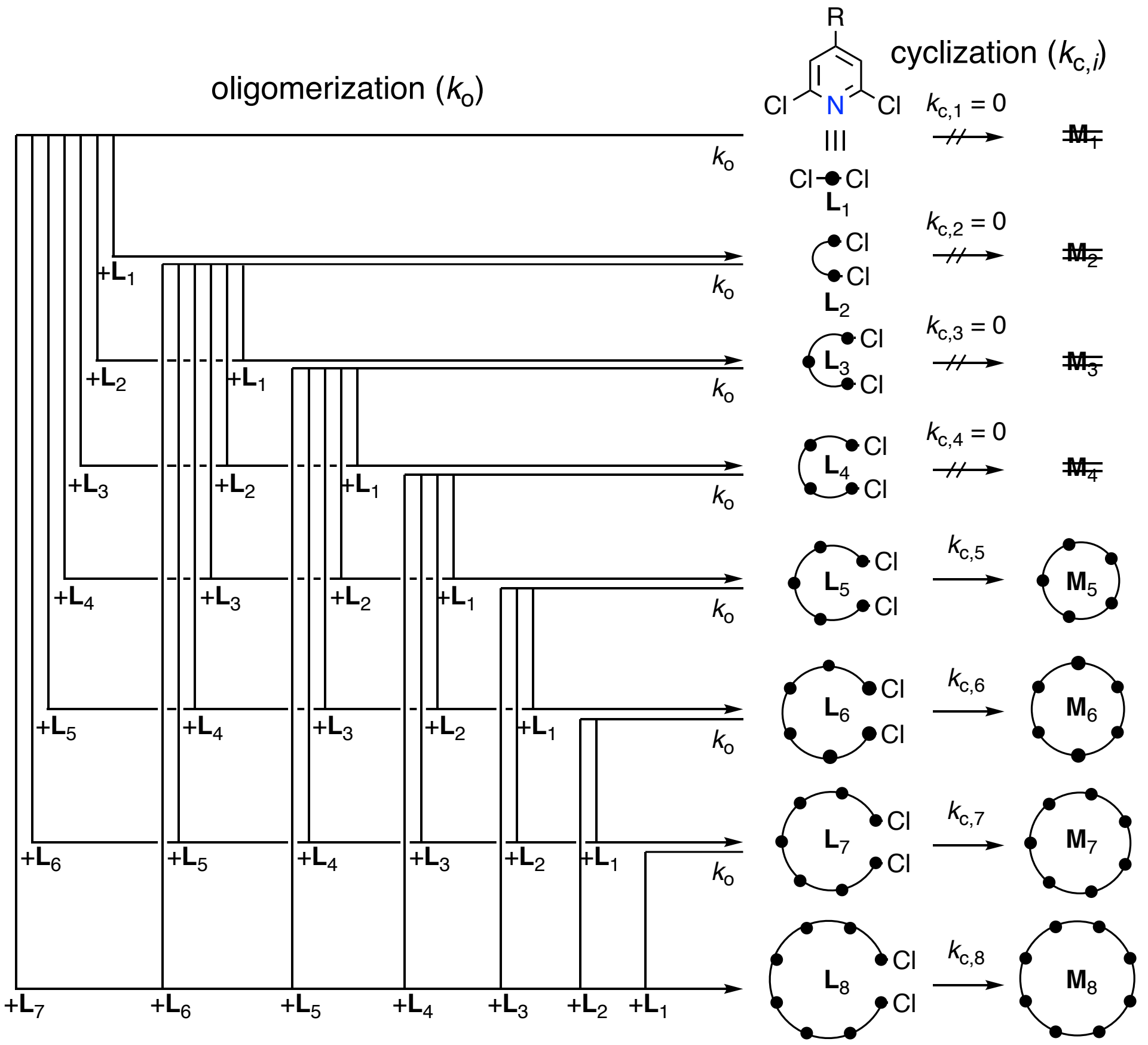

Figure S2. Reaction pathways and rate constants for k-model **a**

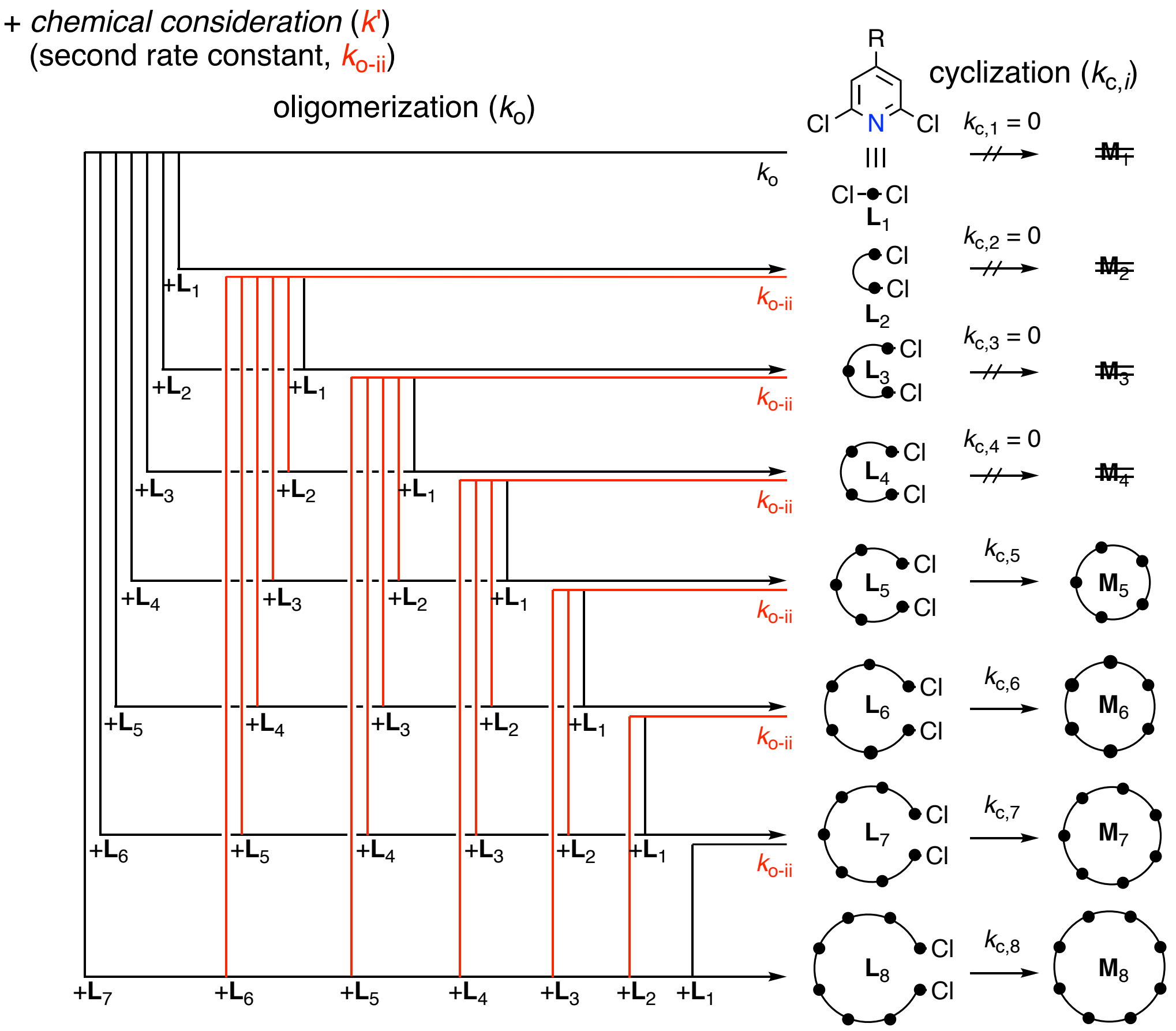

Figure S3. Reaction pathways and rate constants for *k*-model **b** with chemical considerations

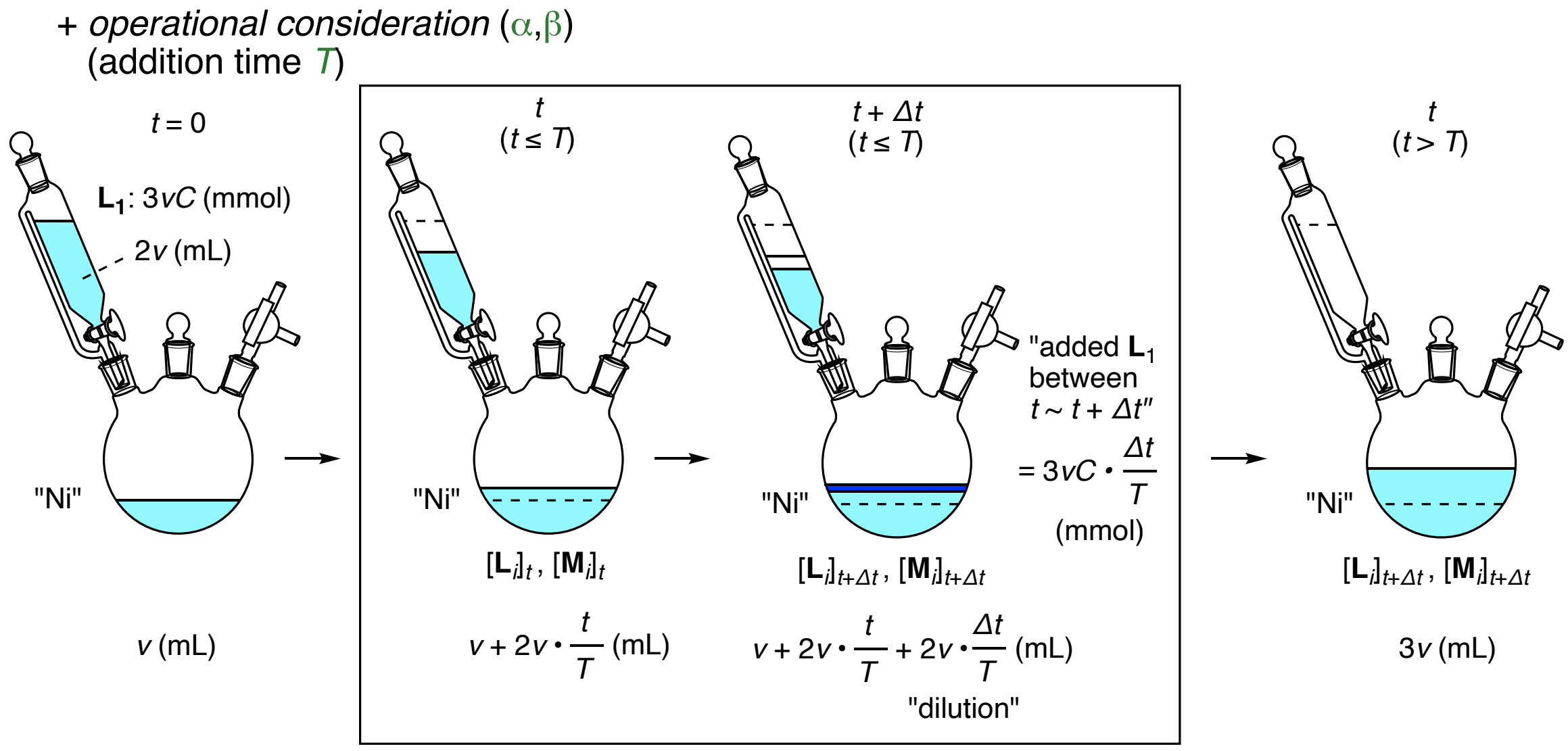

"Dilution of the reaction mixture"

$$\alpha = \frac{v + 2v \cdot \frac{t}{T}}{v + 2v \cdot \frac{t}{T} + 2v \cdot \frac{\Delta t}{T}} = \frac{1 + \frac{2t}{T}}{1 + \frac{2(t + \Delta t)}{T}}$$

"Increase of the monomer concentration [L_1]"

$$\beta = \frac{3vC \cdot \frac{\Delta t}{T}}{v + 2v \cdot \frac{t}{T} + 2v \cdot \frac{\Delta t}{T}} = \frac{3C \cdot \frac{\Delta t}{T}}{1 + \frac{2(t + \Delta t)}{T}}$$

Figure S4. Factors taken into account for operational considerations

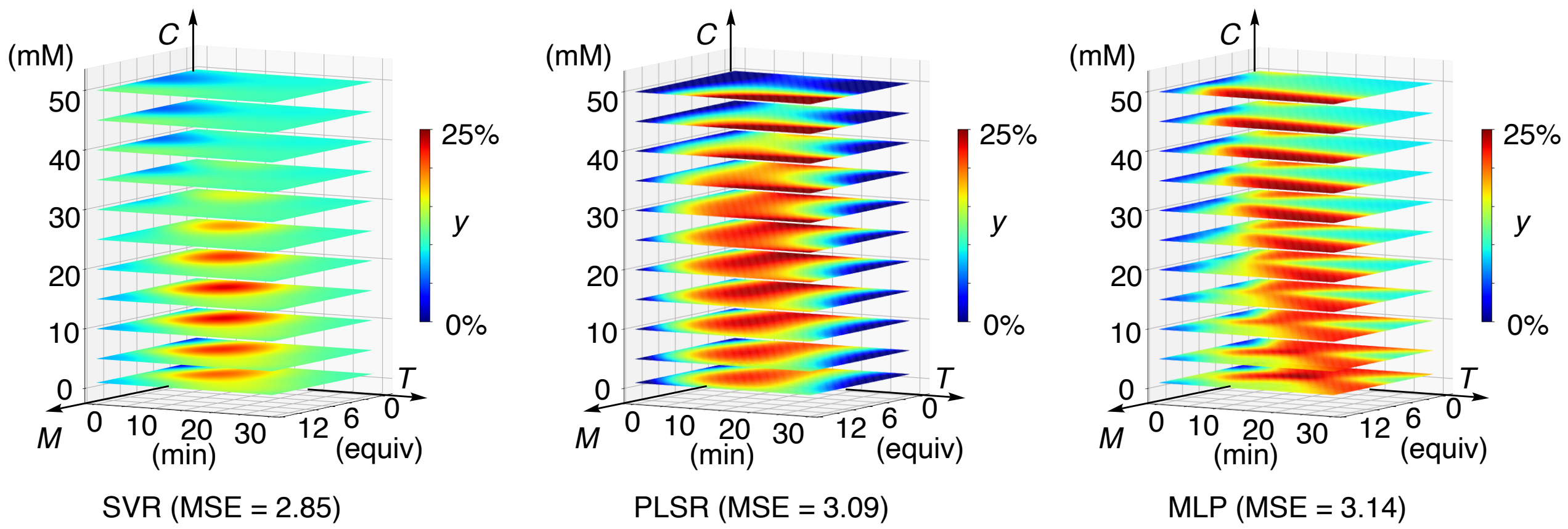

Figure S5. Yield distribution models (*y*-models) for $\mathbf{4}_5$. The ML methods and MSE values are shown under the heatmaps.

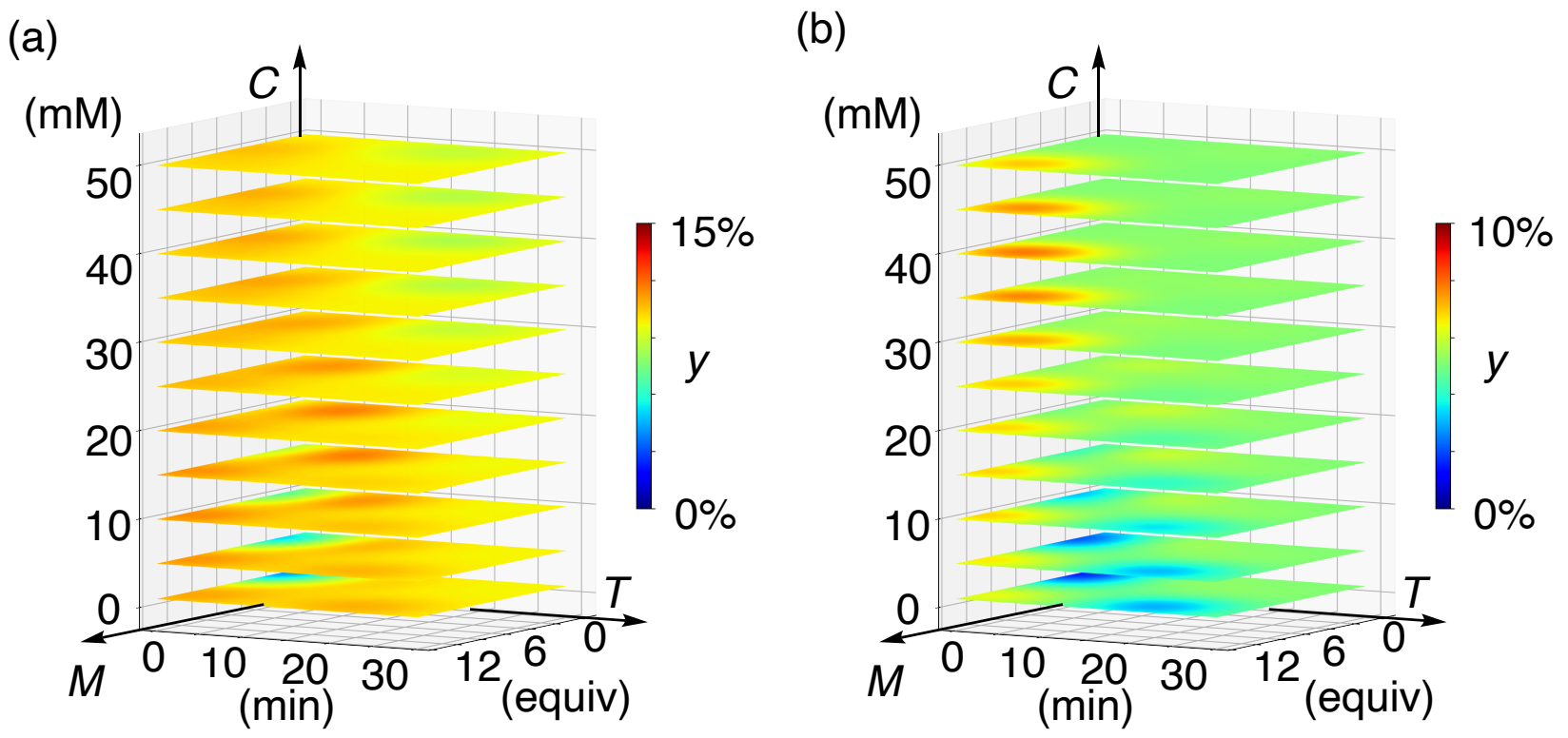

Figure S6. Yield distribution models (y-models) for $\mathbf{4}_i$. (a) i = 6. (b) i = 7.

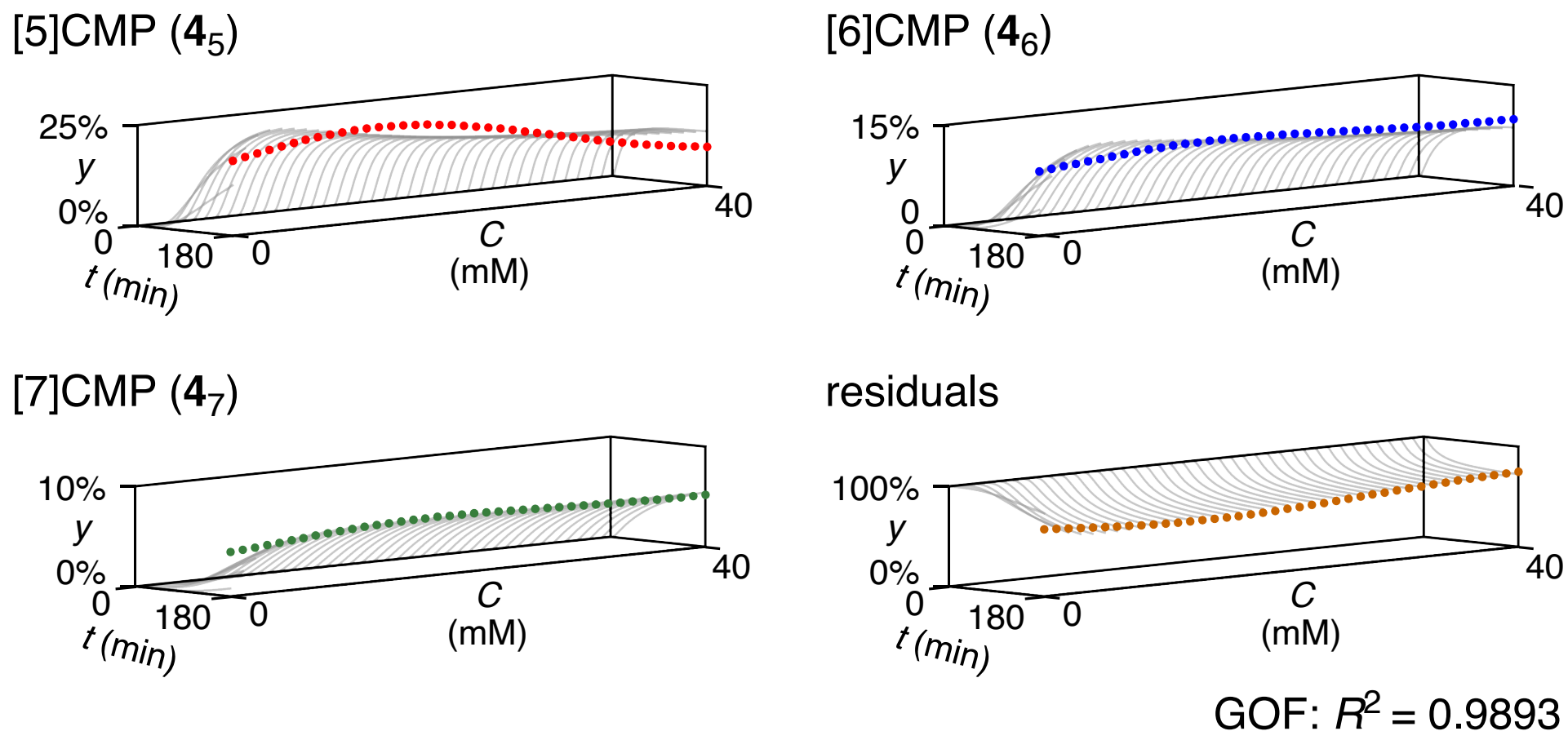

Figure S7. Regression curves for [i]CMP $\mathbf{4}_i$ drawn by the Euler-formulated rate equation after convergence

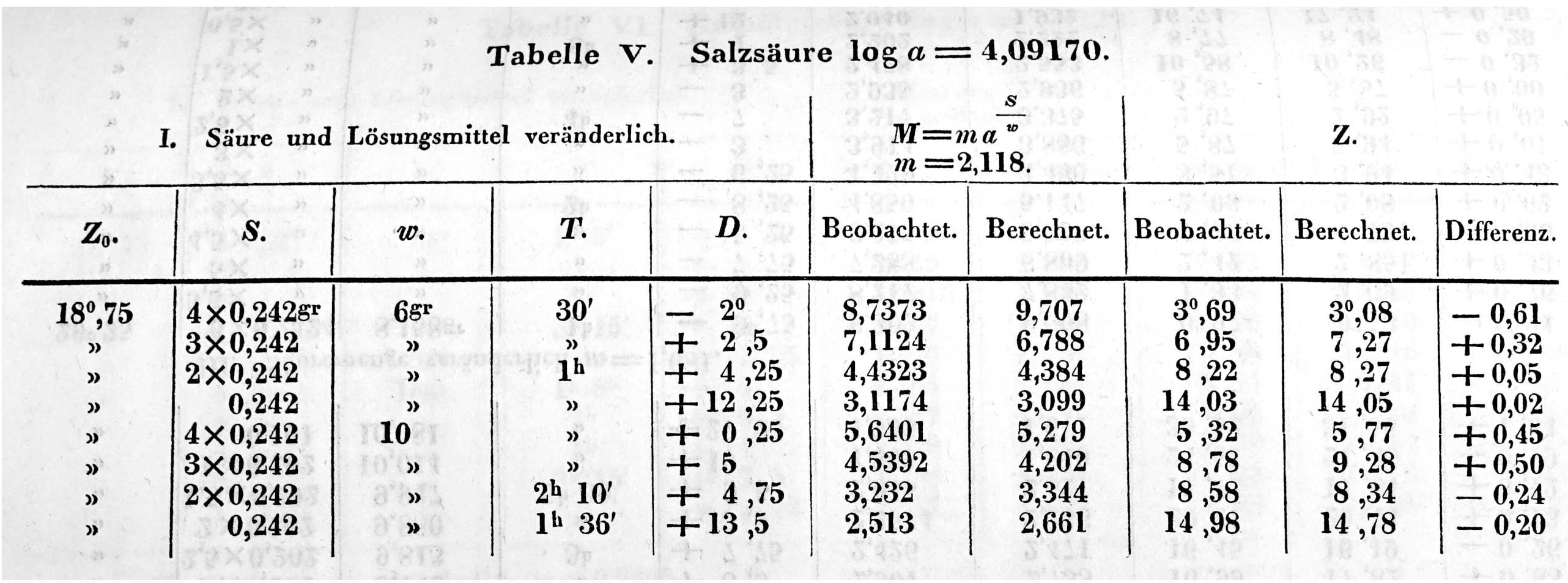

Tabelle V. Salzsäure log a = 4,09170.

I. Säure und Lösungsmittel veränderlich.

Z_0.	S.	w.	T.	D.	$M = m a^{\frac{s}{w}}$, m = 2,118. Beobachtet.	Berechnet.	Z. Beobachtet.	Berechnet.	Differenz.
18°,75	4×0,242gr	6gr	30′	— 2°	8,7373	9,707	3°,69	3°,08	— 0,61
»	3×0,242	»	»	+ 2,5	7,1124	6,788	6,95	7,27	+ 0,32
»	2×0,242	»	1h	+ 4,25	4,4323	4,384	8,22	8,27	+ 0,05
»	0,242	»	»	+ 12,25	3,1174	3,099	14,03	14,05	+ 0,02
»	4×0,242	10	»	+ 0,25	5,6401	5,279	5,32	5,77	+ 0,45
»	3×0,242	»	»	+ 5	4,5392	4,202	8,78	9,28	+ 0,50
»	2×0,242	»	2h 10′	+ 4,75	3,232	3,344	8,58	8,34	— 0,24
»	0,242	»	1h 36′	+ 13,5	2,513	2,661	14,98	14,78	— 0,20

Figure S8. Reanalyzed data (section I, Table V) reported in Wilhelmy's 1850 paper [23] (photograph taken by the authors)

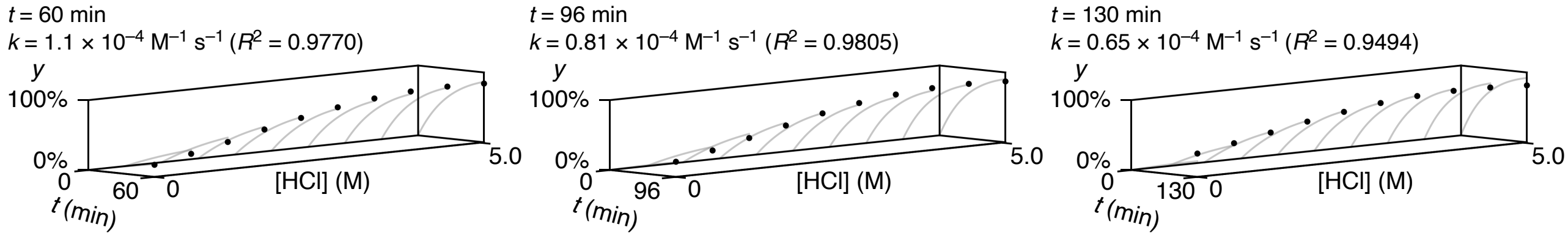

Figure S9. Regression curves for HCl-catalyzed synthesis of invert sugar at different reaction times

Supplementary Tables

Table S1. Experimental yield datasets for $NiCl_2$•COPy-5

	yield of $\mathbf{2}_5$ (%)		
(M,T,C)	1st run	2nd run	3rd run
(5,1,1)	7.1	2.6	4.4
(5,5,10)	22	18	16
(5,20,40)	43	48	49
(10,5,1)	10	5.9	10
(10,20,10)	30	27	27
(10,1,40)	19	16	20
(20,20,1)	8.2	7.4	10
(20,1,10)	15	15	13
(20,5,40)	31	29	27

Table S2. Concentration-dependent yield datasets for $\mathbf{2}_5$@(M,T,C) = (4,20,C) from SVR y-model

C (mM)				yield of $\mathbf{2}_5$ (%)				yield of residuals (%) [= 100–(yield of $\mathbf{2}_5$)]			
1	2	3	4	28.1	28.8	29.6	30.3	71.9	71.2	70.4	69.7
5	6	7	8	31.1	31.8	32.6	33.3	68.9	68.2	67.4	66.7
9	10	11	12	34.1	34.9	35.6	36.4	65.9	65.1	64.4	63.6
13	14	15	16	37.1	37.8	38.5	39.2	62.9	62.2	61.5	60.8
17	18	19	20	39.9	40.5	41.2	41.8	60.1	59.5	58.8	58.2
21	22	23	24	42.3	42.9	43.4	43.9	57.7	57.1	56.6	56.1
25	26	27	28	44.3	44.7	45.1	45.5	55.7	55.3	54.9	54.5
29	30	31	32	45.8	46.0	46.2	46.4	54.2	54.0	53.8	53.6
33	34	35	36	46.6	46.7	46.7	46.7	53.4	53.3	53.3	53.3
37	38	39	40	46.7	46.6	46.5	46.4	53.3	53.4	53.5	53.6

Table S3. Raw data for Akaike's information criterion of k-models

k-model	N	k	SS	AIC	w_i
**a**	80	5	1723	255.6	5.11×10^{-43}
**b**	80	6	1565	249.9	8.99×10^{-42}
**c**	80	5	1698	254.4	9.15×10^{-43}
**d**	80	6	147	60.8	1

Table S4. Experimental yield datasets for [i]CMP

(M,T,C)	yield of [i]CMP ($\mathbf{4}_i$) (%)								
	1st run			2nd run			3rd run		
	[5]CMP	[6]CMP	[7]CMP	[5]CMP	[6]CMP	[7]CMP	[5]CMP	[6]CMP	[7]CMP
(2,1,1)	2.9	3.2	1.2	4.7	2.6	2.2	2.1	2.8	1.1
(2,5,10)	17	16	4.3	20	8.1	5.2	19	11	4.7
(2,20,40)	8.4	10	6.4	10	6.3	5.9	8.7	7.5	4.5
(5,5,1)	17	12	4.0	16	8.1	3.2	20	7.5	4.0
(5,20,10)	15	11	5.2	14	11	5.8	15	8.5	4.7
(5,1,40)	5.3	13	5.4	5.0	11	4.7	6.0	12	5.3
(10,20,1)	17	12	3.0	16	13	3.5	14	8.7	2.4
(10,1,10)	8.3	14	6.9	8.8	13	8.2	4.9	7.1	6.2
(10,5,40)	12	12	8.4	11	13	8.2	14	8.9	7.2

Table S5. Concentration-dependent yield datasets for $\mathbf{4}_i$@(M,T,C) = (3,9,C) from SVR y-model

C (mM)	yield of $\mathbf{4}_5$ (%)	yield of $\mathbf{4}_6$ (%)	yield of $\mathbf{4}_7$ (%)	yield of residuals (%) [= 100–(yield of $\mathbf{4}_5$ & $\mathbf{4}_6$ & $\mathbf{4}_7$)]
1 2 3 4	18.6 19.3 19.9 20.4	9.7 9.8 10.0 10.2	4.5 4.6 4.7 4.8	67.2 66.4 65.5 64.6
5 6 7 8	21.0 21.5 21.9 22.3	10.3 10.5 10.7 10.9	4.9 5.0 5.1 5.2	63.8 63.0 62.3 61.6
9 10 11 12	22.6 22.9 23.1 23.2	11.0 11.1 11.3 11.4	5.3 5.4 5.5 5.6	61.1 60.6 60.2 59.9
13 14 15 16	23.2 23.2 23.0 22.8	11.4 11.5 11.5 11.5	5.6 5.7 5.7 5.8	59.7 59.7 59.7 59.9
17 18 19 20	22.6 22.2 21.8 21.4	11.5 11.5 11.4 11.4	5.8 5.8 5.8 5.8	60.1 60.5 60.9 61.5
21 22 23 24	20.8 20.3 19.7 19.0	11.3 11.2 11.1 11.0	5.8 5.7 5.7 5.7	62.1 62.8 63.5 64.3
25 26 27 28	18.4 17.7 17.0 16.3	10.9 10.8 10.8 10.7	5.6 5.6 5.6 5.5	65.1 65.9 66.7 67.5
29 30 31 32	15.6 14.9 14.3 13.6	10.6 10.5 10.4 10.3	5.5 5.4 5.4 5.4	68.3 69.1 69.9 70.7
33 34 35 36	13.0 12.4 11.9 11.4	10.3 10.2 10.2 10.1	5.3 5.3 5.3 5.3	71.4 72.0 72.7 73.2
37 38 39 40	10.9 10.5 10.1 9.8	10.1 10.0 10.0 10.0	5.2 5.2 5.2 5.2	73.8 74.2 74.7 75.1

Table S6. Yield datasets converted to modern notation from Wilhelmy's paper [23]

[HCl] (M)	t (min)	yield of invert sugar **6**	yield of sucrose **5**
4.42	30	80.3%	19.7%
3.32	30	62.9%	37.1%
2.21	60	56.2%	44.8%
1.11	60	25.2%	74.8%
2.65	60	71.6%	28.4%
1.99	60	53.2%	46.8%
1.33	130	54.2%	45.8%
0.66	96	20.1%	79.9%

Table S7. Concentration-dependent yield datasets for invert sugar from SVR *y*-model (t = 30 min)

[HCl] (M)	yield of **6** (%)	yield of **5** (%)
0.5	21.4	78.6
1.0	28.7	71.3
1.5	37.5	62.5
2.0	46.8	53.2
2.5	55.9	44.1
3.0	63.7	36.3
3.5	69.8	30.2
4.0	73.7	26.3
4.5	75.6	24.4
5.0	75.5	24.5

Table S8. Concentration-dependent yield datasets for invert sugar from SVR *y*-model (t = 60 min)

[HCl] (M)	yield of **6** (%)	yield of **5** (%)
0.5	17.7	82.3
1.0	27.6	72.4
1.5	39.2	60.8
2.0	51.3	48.7
2.5	62.8	37.2
3.0	72.4	27.6
3.5	79.6	20.4
4.0	83.9	16.1
4.5	85.4	14.6
5.0	84.5	15.5

Table S9. Concentration-dependent yield datasets for invert sugar from SVR *y*-model (t = 96 min)

[HCl] (M)	yield of **6** (%)	yield of **5** (%)
0.5	21.8	78.2
1.0	32.6	67.4
1.5	44.8	55.2
2.0	57.4	42.6
2.5	68.9	31.1
3.0	78.4	21.6
3.5	85.0	15.0
4.0	88.6	11.4
4.5	89.2	10.8
5.0	87.2	12.8

Table S10. Concentration-dependent yield datasets for invert sugar from SVR *y*-model (t = 130 min)

[HCl] (M)	yield of **6** (%)	yield of **5** (%)
0.5	33.7	66.3
1.0	42.8	57.2
1.5	52.8	47.2
2.0	62.7	37.3
2.5	71.5	28.5
3.0	78.4	21.6
3.5	82.8	17.2
4.0	84.7	15.3
4.5	84.1	15.9
5.0	81.7	18.3

Supplementary Equations

k-model **a**:

$$[\mathbf{L}_i]_{t+\Delta t} = [\mathbf{L}_i]_t + \Delta t \cdot k_\mathrm{o} \sum_{j=1}^{i-1} [\mathbf{L}_j]_t [\mathbf{L}_{i-j}]_t - \left(\Delta t \cdot 2k_\mathrm{o} [\mathbf{L}_i]_t \sum_{j=1}^{8-i} [\mathbf{L}_j]_t + \Delta t \cdot k_{\mathrm{c},i} [\mathbf{L}_i]_t \right) \tag{S1}$$

$$[\mathbf{M}_i]_{t+\Delta t} = [\mathbf{M}_i]_t + \Delta t \cdot k_{\mathrm{c},i} [\mathbf{L}_i]_t \tag{S2}$$

k-model **b**:

$$\begin{aligned}[\mathbf{L}_i]_{t+\Delta t} = [\mathbf{L}_i]_t &+ \Delta t \cdot k_\mathrm{o} \sum_{j=1,i-1} [\mathbf{L}_j]_t [\mathbf{L}_{i-j}]_t + \Delta t \cdot k_{\mathrm{o-ii}} \sum_{j=2}^{i-2} [\mathbf{L}_j]_t [\mathbf{L}_{i-j}]_t \\ &- \left(\Delta t \cdot 2k_\mathrm{o} [\mathbf{L}_i]_t [\mathbf{L}_1]_t + \Delta t \cdot 2k_{\mathrm{o-ii}} [\mathbf{L}_i]_t \sum_{j=2}^{8-i} [\mathbf{L}_j]_t + \Delta t \cdot k_{\mathrm{c},i} [\mathbf{L}_i]_t \right)\end{aligned} \tag{S3}$$

+ eq. S2 for $[\mathbf{M}_i]_{t+\Delta t}$

k-model **c**:

$t \leq T$

$$[\mathbf{L}_1]_{t+\Delta t} = \frac{1+\frac{2t}{T}}{1+\frac{2(t+\Delta t)}{T}}\left([\mathbf{L}_1]_t - \Delta t \cdot 2k_{\mathrm{o}}[\mathbf{L}_1]_t \sum_{j=1}^{7}[\mathbf{L}_j]_t\right) + \frac{\frac{3C\Delta t}{T}}{1+\frac{2(t+\Delta t)}{T}} \tag{S4}$$

$$[\mathbf{L}_i]_{t+\Delta t} = \frac{1+\frac{2t}{T}}{1+\frac{2(t+\Delta t)}{T}}\left([\mathbf{L}_i]_t + \Delta t \cdot k_{\mathrm{o}} \sum_{j=1}^{i-1}[\mathbf{L}_j]_t[\mathbf{L}_{i-j}]_t - \left(\Delta t \cdot 2k_{\mathrm{o}}[\mathbf{L}_i]_t \sum_{j=1}^{8-i}[\mathbf{L}_j]_t + \Delta t \cdot k_{\mathrm{c},i}[\mathbf{L}_i]_t\right)\right) \tag{S5}$$

$$[\mathbf{M}_i]_{t+\Delta t} = \frac{1+\frac{2t}{T}}{1+\frac{2(t+\Delta t)}{T}}\left([\mathbf{M}_i]_t + \Delta t \cdot k_{\mathrm{c},i}[\mathbf{L}_i]_t\right) \tag{S6}$$

$t > T$

+ eq. S1 for $[\mathbf{L}_i]_{t+\Delta t}$ and eq. S2 for $[\mathbf{M}_i]_{t+\Delta t}$

k-model **d**:

$t \leq T$

eq. S4 for $[\mathbf{L}_1]_{t+\Delta t}$

$$\begin{aligned}[\mathbf{L}_i]_{t+\Delta t} = \frac{1+\frac{2t}{T}}{1+\frac{2(t+\Delta t)}{T}}\Bigg([\mathbf{L}_i]_t &+ \Delta t \cdot k_{\mathrm{o}} \sum_{j=1,i-1}[\mathbf{L}_j]_t[\mathbf{L}_{i-j}]_t + \Delta t \cdot k_{\mathrm{o-ii}} \sum_{j=2}^{i-2}[\mathbf{L}_j]_t[\mathbf{L}_{i-j}]_t \\ &- \left(\Delta t \cdot 2k_{\mathrm{o}}[\mathbf{L}_1]_t[\mathbf{L}_i]_t + \Delta t \cdot 2k_{\mathrm{o-ii}}[\mathbf{L}_i]_t \sum_{j=2}^{8-i}[\mathbf{L}_j]_t + \Delta t \cdot k_{\mathrm{c},i}[\mathbf{L}_i]_t\right)\Bigg)\end{aligned} \tag{S7}$$

eq. S6 for $[\mathbf{M}_i]_{t+\Delta t}$

$t > T$

+ eq. S3 for $[\mathbf{L}_i]_{t+\Delta t}$ and eq. S2 for $[\mathbf{M}_i]_{t+\Delta t}$

k-model for invert sugar:

$$[\mathbf{6}]_{t+\Delta t} = [\mathbf{6}]_t + \Delta t \cdot k[\mathbf{5}]_t[\mathrm{HCl}]_t \tag{S8}$$

$$[\mathbf{5}]_{t+\Delta t} = [\mathbf{5}]_t - \Delta t \cdot k[\mathbf{5}]_t[\mathrm{HCl}]_t \tag{S9}$$

Supplementary Codes

Code S1. Grid search for hyperparameters for the SVR model (*y*-model for $NiCl_2$•COPy-5)

```
# Importing libraries
import pandas as pd
from sklearn import svm
from sklearn.model_selection import GridSearchCV, LeaveOneOut
from sklearn.preprocessing import StandardScaler
import matplotlib.pyplot as plt
from sklearn.metrics import r2_score

# Loading experimental data
df = pd.read_csv("FILENAME.csv").dropna()     #to be modified for other files
names = ('m', 't', 'c')
X = df.loc[:, names].to_numpy()
y = df['y'].to_numpy()

# Pre-normalize the input X
scaler = StandardScaler()
X_scaled = scaler.fit_transform(X)

# Ranges of hyperparameters for optimization
svm_params = {
    "C": [1*i for i in range(1, 51)],         #to be modified for other models
    "epsilon": [0.1*i for i in range(1, 21)], #to be modified for other models
    "gamma": [0.1*i for i in range(1, 21)]    #to be modified for other models

}

# Grid search with LOOCV.
scoring = {
    "MSE": "neg_mean_squared_error",
    "MAPE": "neg_mean_absolute_percentage_error"
}

grid_search = GridSearchCV(
    svm.SVR(),
    svm_params,
    cv=LeaveOneOut(),
    scoring=scoring,
    refit="MSE",
    n_jobs=-1
)

grid_search.fit(X_scaled, y)

print(f"Best model params: {grid_search.best_params_}")

mse = -grid_search.cv_results_["mean_test_MSE"][grid_search.best_index_]
mape = -grid_search.cv_results_["mean_test_MAPE"][grid_search.best_index_] * 100

print(f"LOOCV MSE: {mse:.6f}")
print(f"LOOCV MAPE: {mape:.2f} %")

# Constructing a SVR model with the optimized parameters
bestestimator = svm.SVR(**grid_search.best_params_)
bestestimator.fit(X_scaled, y)
pred = bestestimator.predict(X_scaled)

# Plotting predicted values against experimental ones
fig, ax1 = plt.subplots(1, 1, clear=True, figsize=(5, 4))
plt.scatter(y, pred, color='black')
plt.plot([-1, 100], [-1, 100], ls="--", c=".3")
ax1.set_ylabel('ML-derived yield (%)')
ax1.set_xlabel('Experimental yield (%)')
plt.tight_layout()
plt.show()
```

```
print(f"r2 {r2_score(y, pred)}")
```

Code S2. Generating SVR heatmap and concentration-dependent yield data at the hit (*y*-model for $NiCl_2$•COPy-5)

```
import numpy as np
import pandas as pd
import matplotlib
import matplotlib.pyplot as plt
from sklearn import svm
from sklearn.pipeline import Pipeline
from sklearn.preprocessing import StandardScaler

def main():
    # Loading data
    df = pd.read_csv("FILENAME.csv")  #to be modified for other files
    df = df.dropna()

    names = ("m", "t", "c")
    X = df.loc[:, names]
    y = df["y"]

    # SVR regression
    reg_y_svr = Pipeline([
        ("scl", StandardScaler()),
        ("clf", svm.SVR(kernel="rbf", C=49, epsilon=1.3, gamma=0.2))  #to be modified for other models
    ])

    reg_y_svr.fit(X, y)

    print("Showing SVR results")
    draw_heatmap(reg_y_svr)

    print("Writing CSV")
    output_c_dependent_csv(reg_y_svr)

# Drawing 3D heatmap
def draw_heatmap(regressor):
    fig = plt.figure(figsize=(10, 10))
    ax = fig.add_subplot(111, projection="3d", proj_type="ortho")
    ax.set_box_aspect((1, 1, 1.5))

    x_len, y_len = 50, 50

    xs = np.linspace(0, 12, x_len)
    ys = np.linspace(0, 30, y_len)
    z_values = np.array([1, 5, 10, 15, 20, 25, 30, 35, 40, 45, 50])

    norm = matplotlib.colors.Normalize(vmin=0, vmax=60)
    cmap = matplotlib.colormaps["jet"]

    for z in z_values:
        xm, ym = np.meshgrid(xs, ys)
        zm = z * np.ones_like(xm)
        r = np.c_[xm.flatten(), ym.flatten(), zm.flatten()]

        pred = regressor.predict(r).reshape(xm.shape)
        clr = cmap(norm(pred))

        ax.plot_surface(
            xm, ym, zm,
            rstride=1,
            cstride=1,
            linewidth=0.2,
            facecolors=clr,
            shade=False
        )
```

```
    ax.set_xlabel("eq.")
    ax.set_ylabel("min")
    ax.set_zlabel("mM")

    ax.set_xlim(-1, 12)
    ax.set_ylim(-1, 31)

    ax.view_init(elev=7, azim=30)

    plt.show()

def output_c_dependent_csv(regressor):
    m_fixed = 4
    t_fixed = 20
    c_values = np.linspace(1, 40, 40)

    X_pred = pd.DataFrame({
        "m": np.full_like(c_values, m_fixed, dtype=float),
        "t": np.full_like(c_values, t_fixed, dtype=float),
        "c": c_values
    })

    y_pred = regressor.predict(X_pred)

    out = pd.DataFrame({
        "c": c_values,
        "y_5": y_pred,
        "y_R": 100 - y_pred
    })

    out.to_csv("C-dependent_y.csv", index=False)

    print(out)
    print("Saved: C-dependent_y.csv")

main()
```

Code S3. Least-squares regression by SciPy

```
import time
import numpy as np
import xlwings as xw
from scipy.optimize import minimize

# ========= Setting =========
WORKBOOK_PATH = "FILENAME.xlsx"      #to be modified for other files
MAIN_SHEET_NAME = "CYAN regression"

TARGET_ADDR = "B53"
VAR_RANGE_ADDR = "B2:B7"             #to be modified for other models
CONSTRAINT_MIN_ADDR = "B57"
CONSTRAINT_MAX_ADDR = "B58"
CONSTRAINT_MIN_LIMIT = 0
CONSTRAINT_MAX_LIMIT = 100
VAR_LOWER_BOUND = 0.0
VAR_UPPER_BOUND = None
METHOD = "SLSQP"
MAX_ITER = 1000
TOL = 1e-5
RECALC_SLEEP_SEC = 0.0
PENALTY = 1e30
LOG_EVERY = 25
# =======================


def to_float_or_penalty(v):
    try:
        if v is None:
            return PENALTY
```

```
        if isinstance(v, str):
            s = v.strip()
            if s == "" or s.startswith("#"):
                return PENALTY
            v = float(s)

        f = float(v)

        if not np.isfinite(f):
            return PENALTY

        return f

    except Exception:
        return PENALTY


def main():
    app = xw.App(visible=True)
    app.screen_updating = False
    app.display_alerts = False

    try:
        app.calculation = "automatic"
    except Exception:
        pass

    try:
        wb = app.books.open(WORKBOOK_PATH)
        ws = wb.sheets[MAIN_SHEET_NAME]

        x0 = np.array(
            ws.range(VAR_RANGE_ADDR)
            .options(np.array, dtype=float)
            .value
        ).reshape(-1)

        n = x0.size
        print(f"Initial x0 ({n} vars) = {x0}")

        bounds = [
            (VAR_LOWER_BOUND, VAR_UPPER_BOUND)
            for _ in range(n)
        ]

        cache = {
            "x": None,
            "f": None,
            "cmin": None,
            "cmax": None,
        }

        state = {
            "k": 0,

            "best_f": np.inf,
            "best_x": x0.copy(),

            "best_feasible_f": np.inf,
            "best_feasible_x": None,

            "bad_count": 0,
        }

        def evaluate_once(x):
            x = np.array(x, dtype=float).reshape(-1)

            if (
                cache["x"] is not None
```

```
        and np.allclose(x, cache["x"], rtol=0, atol=1e-12)
    ):
        return cache["f"], cache["cmin"], cache["cmax"]

    ws.range(VAR_RANGE_ADDR).value = x.reshape(-1, 1)
    wb.app.calculate()

    if RECALC_SLEEP_SEC > 0:
        time.sleep(RECALC_SLEEP_SEC)

    f = to_float_or_penalty(ws.range(TARGET_ADDR).value)
    cmin = to_float_or_penalty(ws.range(CONSTRAINT_MIN_ADDR).value)
    cmax = to_float_or_penalty(ws.range(CONSTRAINT_MAX_ADDR).value)

    cache["x"] = x.copy()
    cache["f"] = f
    cache["cmin"] = cmin
    cache["cmax"] = cmax

    return f, cmin, cmax

def objective(x):
    f, cmin, cmax = evaluate_once(x)

    state["k"] += 1

    if f >= PENALTY:
        state["bad_count"] += 1
        print(f"[{state['k']}] f = PENALTY, x = {x}")
        return f

    feasible = (
        cmin >= CONSTRAINT_MIN_LIMIT
        and cmax <= CONSTRAINT_MAX_LIMIT
    )

    if f < state["best_f"]:
        state["best_f"] = f
        state["best_x"] = np.array(x, dtype=float).copy()

    if feasible and f < state["best_feasible_f"]:
        state["best_feasible_f"] = f
        state["best_feasible_x"] = np.array(x, dtype=float).copy()

        print(
            f"[{state['k']}] best feasible f = "
            f"{state['best_feasible_f']:.12g}, "
            f"B57 = {cmin:.12g}, "
            f"B58 = {cmax:.12g}, "
            f"x = {state['best_feasible_x']}"
        )

    elif state["k"] % LOG_EVERY == 0:
        print(
            f"[{state['k']}] f = {f:.12g}, "
            f"B57 = {cmin:.12g}, "
            f"B58 = {cmax:.12g}, "
            f"feasible = {feasible}"
        )

    return f

def constraint_lower(x):
    f, cmin, cmax = evaluate_once(x)

    if not np.isfinite(cmin):
        return -1e10

    return cmin - CONSTRAINT_MIN_LIMIT
```

```
    def constraint_upper(x):
        f, cmin, cmax = evaluate_once(x)

        if not np.isfinite(cmax):
            return -1e10

        return CONSTRAINT_MAX_LIMIT - cmax

    constraints = [
        {"type": "ineq", "fun": constraint_lower},
        {"type": "ineq", "fun": constraint_upper},
    ]

    print("¥n=== Start optimization ===")

    res = minimize(
        objective,
        x0,
        method=METHOD,
        bounds=bounds,
        constraints=constraints,
        options={
            "maxiter": MAX_ITER,
            "ftol": TOL,
            "disp": True,
        },
    )

    print("¥n=== SciPy Result ===")
    print(res)
    print("SciPy success =", res.success)
    print("SciPy message =", res.message)

    if state["best_feasible_x"] is not None:
        final_x = state["best_feasible_x"]
        print("¥nUsing best feasible solution.")
    else:
        final_x = np.array(res.x, dtype=float).copy()
        print("¥nWARNING: No feasible solution was found during optimization.")
        print("Using scipy final x instead.")

    ws.range(VAR_RANGE_ADDR).value = final_x.reshape(-1, 1)
    wb.app.calculate()

    final_f = ws.range(TARGET_ADDR).value
    final_cmin = ws.range(CONSTRAINT_MIN_ADDR).value
    final_cmax = ws.range(CONSTRAINT_MAX_ADDR).value

    print("¥n=== Final Check ===")
    print("Objective B53 =", final_f)
    print("Constraint B57 =", final_cmin)
    print("Constraint B58 =", final_cmax)
    print("Required B57 >=", CONSTRAINT_MIN_LIMIT)
    print("Required B58 <=", CONSTRAINT_MAX_LIMIT)

    print("¥n=== Best values ===")
    print("Best feasible f =", state["best_feasible_f"])
    print("Best feasible x =", state["best_feasible_x"])
    print("Best unconstrained f =", state["best_f"])
    print("Best unconstrained x =", state["best_x"])
    print("Total evals =", state["k"])
    print("Bad evals =", state["bad_count"])

    out_path = WORKBOOK_PATH.replace(".xlsx", "_optimized.xlsx")
    wb.save(out_path)

    print("¥n=== Saved ===")
    print(f"Saved optimized workbook: {out_path}")

finally:
```

```
        app.screen_updating = True
        app.display_alerts = True

if __name__ == "__main__":
    main()
```

Code S4. Generation of CYAN curves

```
from openpyxl import load_workbook
import numpy as np
import matplotlib.pyplot as plt
from mpl_toolkits.mplot3d.art3d import Line3D

filename = "FILENAME.xlsx"  #to be modified for other files

DPI_OUT = 720
T_TARGET = 980              #to be modified for other models
FIGSIZE = (1500 / 360, 1000 / 360)
VIEW_ELEV = 6
VIEW_AZIM = -45
BOX_ASPECT = (0.2, 1, 0.15)
GRAY_LINE = (0.5686, 0.5686, 0.5686, 0.5)

def read_column(ws, col, rows):
    """Read one Excel column as a NumPy float array."""
    return np.array([ws[f"{col}{r}"].value for r in rows], dtype=float)

def setup_3d_axis(ax, times, concs, zlim):
    """Apply common 3D axis settings."""
    ax.set_xlim(times.min(), times.max())
    ax.set_ylim(concs.min(), concs.max())
    ax.set_zlim(*zlim)

    ax.set_proj_type("ortho")
    ax.view_init(elev=VIEW_ELEV, azim=VIEW_AZIM)
    ax.set_box_aspect(BOX_ASPECT)

    ax.xaxis._axinfo["tick"]["color"] = (0, 0, 0, 1)
    ax.yaxis._axinfo["tick"]["color"] = (0, 0, 0, 1)
    ax.zaxis._axinfo["tick"]["color"] = (0, 0, 0, 1)

    ax.tick_params(axis="x", labelsize=12, colors="black", width=0.6, length=10)
    ax.tick_params(axis="y", labelsize=12, colors="black", width=0.6, length=5000)
    ax.tick_params(axis="z", labelsize=12, colors="black", width=0.6, length=10)

    try:
        ax.xaxis.set_pane_color((1, 1, 1, 0))
        ax.yaxis.set_pane_color((1, 1, 1, 0))
        ax.zaxis.set_pane_color((1, 1, 1, 0))
    except Exception:
        pass

    ax.grid(False)
    ax.set_xticks([])
    ax.set_yticks([])
    ax.set_zticks([])

    ax.xaxis.line.set_linewidth(0.6)
    ax.yaxis.line.set_linewidth(0.6)
    ax.zaxis.line.set_linewidth(0.6)


def draw_box_edges(ax):
    """Draw custom 3D box edges."""
    xmin, xmax = ax.get_xlim()
    ymin, ymax = ax.get_ylim()
    zmin, zmax = ax.get_zlim()

    vertices = [
```

```
        (xmin, ymin, zmin),
        (xmax, ymin, zmin),
        (xmin, ymax, zmin),
        (xmax, ymax, zmin),
        (xmin, ymin, zmax),
        (xmax, ymin, zmax),
        (xmin, ymax, zmax),
        (xmax, ymax, zmax),
    ]

    edges = [
        (0, 1), (0, 2), (1, 3), (2, 3),
        (4, 6), (6, 7),
        (0, 4), (2, 6), (3, 7),
    ]

    for i, j in edges:
        x1, y1, z1 = vertices[i]
        x2, y2, z2 = vertices[j]

        ax.add_line(Line3D(
            [x1, x2],
            [y1, y2],
            [z1, z2],
            color="black",
            linewidth=0.6,
        ))


def plot_euler_curves(ax, wb, sheet_names, euler_conc_M, rows_time, value_col):
    """Plot gray Euler curves for all concentration sheets."""
    for cM, sheet_name in zip(euler_conc_M, sheet_names):
        ws = wb[sheet_name]

        t_values = read_column(ws, "A", rows_time)
        y_values = read_column(ws, value_col, rows_time)

        c_mM = cM * 1000.0
        c_values = np.full_like(t_values, c_mM, dtype=float)

        ax.plot(
            t_values,
            c_values,
            y_values,
            color=GRAY_LINE,
            linewidth=0.6,
        )


def make_plot(
    wb,
    concs,
    times,
    marker_values,
    euler_conc_M,
    sheet_names,
    rows_time,
    value_col,
    marker_color,
    zlim,
    output_name,
):
    """Create and save one 3D plot."""
    fig = plt.figure(figsize=FIGSIZE, dpi=DPI_OUT)
    ax = fig.add_subplot(111, projection="3d")

    t_const = np.full_like(concs, T_TARGET, dtype=float)

    ax.scatter(
        t_const,
```

```
        concs,
        marker_values,
        c=marker_color,
        marker="o",
        s=1,
        depthshade=False,
        label=f"{output_name} at t = {T_TARGET} min",
    )

    plot_euler_curves(
        ax=ax,
        wb=wb,
        sheet_names=sheet_names,
        euler_conc_M=euler_conc_M,
        rows_time=rows_time,
        value_col=value_col,
    )

    setup_3d_axis(ax, times, concs, zlim)
    draw_box_edges(ax)

    plt.savefig(filename + output_name + ".png", dpi=DPI_OUT)
    plt.show()


def main():
    wb = load_workbook(filename, data_only=True)
    reg_ws = wb["CYAN regression"]

    rows_conc = range(12, 52)
    rows_time = range(3, T_TARGET + 4)

    concs = read_column(reg_ws, "A", rows_conc)
    y5_final = read_column(reg_ws, "B", rows_conc)
    yR_final = read_column(reg_ws, "C", rows_conc)

    euler_conc_M = np.arange(0.001, 0.040 + 0.001, 0.001)
    sheet_names = [f"Euler@{c:0.3f} M" for c in euler_conc_M]

    first_ws = wb[sheet_names[0]]
    times = read_column(first_ws, "A", rows_time)

    make_plot(
        wb=wb,
        concs=concs,
        times=times,
        marker_values=y5_final,
        euler_conc_M=euler_conc_M,
        sheet_names=sheet_names,
        rows_time=rows_time,
        value_col="S",
        marker_color="purple",
        zlim=(0, 60),
        output_name="Y5",
    )

    make_plot(
        wb=wb,
        concs=concs,
        times=times,
        marker_values=yR_final,
        euler_conc_M=euler_conc_M,
        sheet_names=sheet_names,
        rows_time=rows_time,
        value_col="T",
        marker_color=(0.788, 0.396, 0),
        zlim=(0, 100),
        output_name="YR",
    )
```

```
if __name__ == "__main__":
    main()
```

Code S5. Generation of 2D heatmap for invert sugar synthesis

```
import numpy as np
import pandas as pd
import matplotlib.pyplot as plt
import matplotlib.colors as mcolors
from sklearn.pipeline import Pipeline
from sklearn.preprocessing import StandardScaler
from sklearn import svm

# Loading Wilhelmy's experimental data
df = pd.read_csv("Wilhelmy_hydrolysis.csv")
df = df.dropna()
names = ('HCl', 'time')
variables = df.loc[:, names]


# SVR regression
reg_y_svr = Pipeline([
    ('scl', StandardScaler()),
    ('clf', svm.SVR(kernel='rbf', C=132, epsilon=4.9, gamma=0.1))
    ])

reg_y_svr.fit(variables, df['product'].to_numpy().ravel())
df['pred_y_svr'] = reg_y_svr.predict(variables)


# Drawing 2D heatmap
time_min, time_max = 10, 150
HCl_min, HCl_max = 0.1, 5.0
nx, ny = 200, 200

x = np.linspace(time_min, time_max, nx)
y = np.linspace(HCl_min, HCl_max, ny)
xx, yy = np.meshgrid(x, y)

grid = np.c_[yy.ravel(), xx.ravel()]
z = reg_y_svr.predict(grid).reshape(xx.shape)

fig, ax = plt.subplots(figsize=(6, 5))

levels = np.linspace(0, 100, 101)
norm = mcolors.Normalize(vmin=0, vmax=100)

cmap = plt.get_cmap("jet")
cont = ax.contourf(xx, yy, z, levels=levels, cmap=cmap, norm=norm)
cbar = fig.colorbar(cont, ax=ax, ticks=[0, 20, 40, 60, 80, 100])


# Vertical lines at selected times
time_lines = [30, 60, 96, 130]

for t in time_lines:
    ax.axvline(x=t,
```

```
               color='black',
               linewidth=0.8,
               linestyle='-',
               zorder=6)


# Axis settings
ax.set_xlim(10, 150)
ax.set_ylim(0.1, 5)
ax.set_xticks(np.arange(10, 151, 20))
ax.xaxis.tick_top()
ax.xaxis.set_label_position('top')
ax.set_yticks(np.arange(1, 6, 1))

ax.tick_params(axis='both',
               which='major',
               direction='out',
               length=5,
               labelsize=12)

plt.tight_layout()
plt.show()
```

Supplementary References

34. A. B. Pangborn, M. A. Giardello, R. H. Grubbs, R. K. Rosen, and F. J. Timmers, "Safe and convenient procedure for solvent purification," *Organometallics* 15, (1996): 1518-1520. https://doi.org/10.1021/om9503712.
35. C. R. Harris, K. J. Millman, S. J. van der Walt, R. Gommers, P. Virtanen, D. Cournapeau, E. Wieser, J. Taylor, S. Berg, N. J. Smith, R. Kern, M. Picus, S. Hoyer, M. H. van Kerkwijk, M. Brett, A. Haldane, J. D. del Río, M. Wiebe, P. Peterson, P. Gérard-Marchant, K. Sheppard, T. Reddy, W. Weckesser, H. Abbasi, C. Gohlke, and T. E. Oliphant, "Array programming with NumPy," *Nature* 585, (2020): 357-362. https://doi.org/10.1038/s41586-020-2649-2.
36. W. McKinney, "Data structures for statistical computing in Python," in *Proceedings of the 9th Python in Science Conference*, ed. S. van der Walt, and J. Millman, *SciPy* (2010): 56-61.
37. J. D. Hunter, "Matplotlib: A 2D graphics environment," *Computing in Science & Engineering* 9, (2007): 90-95. https://doi.org/10.1109/MCSE.2007.55.
38. F. Pedregosa, G. Varoquaux, A. Gramfort, V. Michel, B. Thirion, O. Grisel, M. Blondel, P. Prettenhofer, R. Weiss, V. Dubourg, J. Vanderplas, A. Passos, D. Cournapeau, M. Brucher, M. Perrot, and É. Duchesnay, "Scikit-learn: Machine learning in Python," *Journal of Machine Learning Research* 12, (2011): 2825-2830.
39. D. M. Hawkins, S. C. Basak, and D. Mills, "Assessing model fit by cross-validation," *Journal of Chemical Information and Computer Sciences* 43, (2003): 579-586. https://doi.org/10.1021/ci025626i.

40. A. Tropsha, P. Gramatica, and V. Gombar, "The importance of being earnest: Validation is the absolute essential for successful application and interpretation of QSPR models," *QSAR & Combinatorial Science* 22, (2003): 69-77. https://doi.org/10.1002/qsar.200390007.
41. F. Zumstein, *Python for Excel: A Modern Environment for Automation and Data Analysis*, (O'Reilly, 2021).
42. P. Virtanen, R. Gommers, T. E. Oliphant, M. Haberland, T. Reddy, D. Cournapeau, E. Burovski, P. Peterson, W. Weckesser, J. Bright, S. J. van der Walt, M. Brett, J. Wilson, K. J. Millman, N. Mayorov, A. R. J. Nelson, E. Jones, R. Kern, E. Larson, C. J. Carey, I. Polat, Y. Feng, E. W. Moore, J. VanderPlas, D. Laxalde, J. Perktold, R. Cimrman, I. Henriksen, E. A. Quintero, C. R. Harris, A. M. Archibald, A. H. Ribeiro, F. Pedregosa, P. van Mulbregt, and SciPy 1.0 Contributors. "SciPy 1.0: fundamental algorithms for scientific computing in Python," *Nature Methods* 17, (2020): 261-272. https://doi.org/10.1038/s41592-019-0686-2.
43. J. C. Butcher, "A history of Runge-Kutta methods," *Applied Numerical Mathematics* 20, (1996): 247-260. https://doi.org/10.1016/0168-9274(95)00108-5.
